# Pathological conformations of disease mutant Ryanodine Receptors revealed by cryo-EM

**DOI:** 10.1038/s41467-021-21141-3

**Published:** 2021-02-05

**Authors:** Kellie A. Woll, Omid Haji-Ghassemi, Filip Van Petegem

**Affiliations:** University of British Columbia, Department of Biochemistry and Molecular Biology, Life Sciences Centre, Vancouver, BC Canada

**Keywords:** Calcium channels, Cryoelectron microscopy, Single-channel recording, Ventricular fibrillation

## Abstract

Ryanodine Receptors (RyRs) are massive channels that release Ca^2+^ from the endoplasmic and sarcoplasmic reticulum. Hundreds of mutations are linked to malignant hyperthermia (MH), myopathies, and arrhythmias. Here, we explore the first MH mutation identified in humans by providing cryo-EM snapshots of the pig homolog, R615C, showing that it affects an interface between three solenoid regions. We also show the impact of apo-calmodulin (apoCaM) and how it can induce opening by bending of the bridging solenoid, mediated by its N-terminal lobe. For R615C RyR1, apoCaM binding abolishes a pathological ‘intermediate’ conformation, distributing the population to a mixture of open and closed channels, both different from the structure without apoCaM. Comparisons show that the mutation primarily affects the closed state, inducing partial movements linked to channel activation. This shows that disease mutations can cause distinct pathological conformations of the RyR and facilitate channel opening by disrupting interactions between different solenoid regions.

## Abstract (web summary)

Ryanodine Receptors (RyRs) release Ca2+ from the endoplasmic and sarcoplasmic reticulum. Mutations in RyR are linked to malignant hyperthermia (MH), myopathies, and arrhythmias. Here, a collection of cryoEM structures provides insights into the molecular consequences of MHrelated RyR mutation R615C, and how apoCaM opens RyR1.

## Introduction

Ryanodine receptors (RyR) are >2-MDa membrane proteins that allow for the release of Ca^2+^ from the ER and SR, thus mediating a range of Ca^2+^-dependent processes^1^. The human genome encodes three isoforms (RyR1-3) with differential cellular expression. Each forms homotetrameric assemblies of ~5000 residue long subunits. RyRs have mostly been studied in the context of cardiac and skeletal muscle tissue, where they release Ca^2+^ required for muscle contraction. Their importance is underscored by the hundreds of sequence variants that have been linked to disease^2–6^. Mutations in RyR1, the skeletal muscle isoform, can cause malignant hyperthermia (MH), central core disease (CCD), and other myopathies. Sequence variants in RyR2, abundant in cardiac myocytes, are mostly linked to catecholaminergic polymorphic ventricular tachycardia (CPVT), a type of stress-induced cardiac arrhythmia. Most mutations cluster in three or four disease hot spots across the sequence. Despite their different locations, functional investigation shows that the majority of mutations induce a gain-of-function phenotype, with increased opening and/or sensitivity to activating ligands such as caffeine and Ca^2+^. Clinically, the MH phenotype usually manifests during general anesthesia with the mutant RyR1 displaying increased sensitivity to activating volatile anesthetics. Currently, there are very limited treatment options for MH, CCD, and CPVT. Therefore, understanding the pathophysiological mechanism of the disease will provide a sound foundation to improve patient care.

Due to their size, RyRs have been popular targets for investigation through cryo-EM^7–10^. These studies have shown the typical mushroom-shape of the protein. The stalk crosses the ER or SR membrane, with a topology similar to voltage-gated channels. The cap is located entirely in the cytosol and is mostly built up by α-solenoid regions and various globular domains. Studies with activating ligands, including Ca^2+^, ATP, caffeine, and diamide insecticides have shown that the cytosolic cap can undergo distinct conformations, highlighting the allosteric nature of the protein^11,12^.

We sought to answer basic questions around the pathophysiology of RyRs: do disease mutations alter the distribution between previously known conformations, or do they result in distinct pathological conformations? Previous experiments with short peptides have suggested the existence of pathological conformations, induced by disease mutations or abnormal conditions in the cell, resulting in altered interactions between different domains^13–16^. Crystallographic and NMR investigation of RyR fragments also hint at distinct conformations induced by disease mutations^17–22^, but very little is known about the impact of disease mutations on full-length RyRs. With mutations located in different regions, the possibility also exists that different mutations have distinct effects on the structure. In order to obtain a full picture, it is necessary to consider the effect of the mutations in both the open and closed states of the channel.

In this study, we probe the effect of a founder disease-causing mutation, originally identified in pigs with porcine stress syndrome. We show that the R615C mutation induces a distinct pathological conformation through weakening of inter-domain interactions. By virtue of adding Calmodulin, a well-known regulator of RyRs, we are able to obtain detailed comparisons of wild-type and disease mutant RyR1 in both the open and closed states.

## Results

### The R615C mutation in porcine RyR1 affects inter-domain interactions that facilitate channel opening

**Fig. 1:**
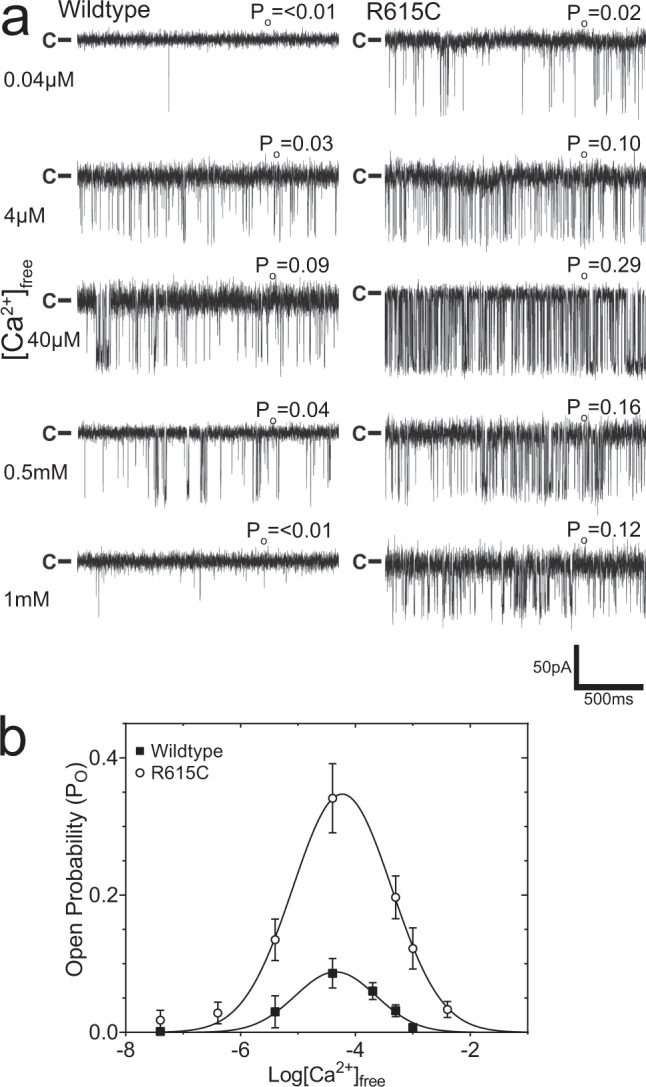
Pig R615C RyR1 has a higher open probability compared to wild type under various calcium (Ca^2+^) concentrations. **a** Representative single-channel traces of WT and R615C pRyR1 at different free Ca^2+^ concentrations. **b** Plots of open probability as a function of free Ca^2+^. Error bars correspond to standard error of the mean (*n* = 6 with each independent recording corresponding to a newly incorporated channel from a different liposome). Single-channel recordings were carried out under symmetrical conditions ([*cis*] = [*trans*]) with a constant −60 mV holding potential.

Several cryo-EM structures of RyRs have been reported, but structural investigations of the pathophysiological conformations are scarce. This is partly due to the difficulty in obtaining large quantities of recombinant RyRs, requiring the majority of investigators to isolate channels from only wild-type (WT) native material^7,8,11,23–26^. To overcome this hurdle, we employed a known homolog to the human R614C MH RyR1 mutation, identified in pigs^4,27^. The homologous pig R615C mutation provided the original link between MH and RyR1 causing a very similar disease state called porcine stress syndrome^28^. We thus capitalized on the availability of homozygous R615C pigs to obtain large quantities of R615C pig RyR1 (pRyR1) for cryo-EM studies, as a representative for N-terminal disease hot spot mutations in RyR1 and RyR2. Figure 1a, b shows single-channel recordings of WT and R615C pRyR1 purified from porcine tissue, indicating a clear gain-of-function for R615C with increased maximum open probability (P_o_) and enhanced sensitivity to Ca^2+^, aligning with previous reports^29^.

**Fig. 2:**
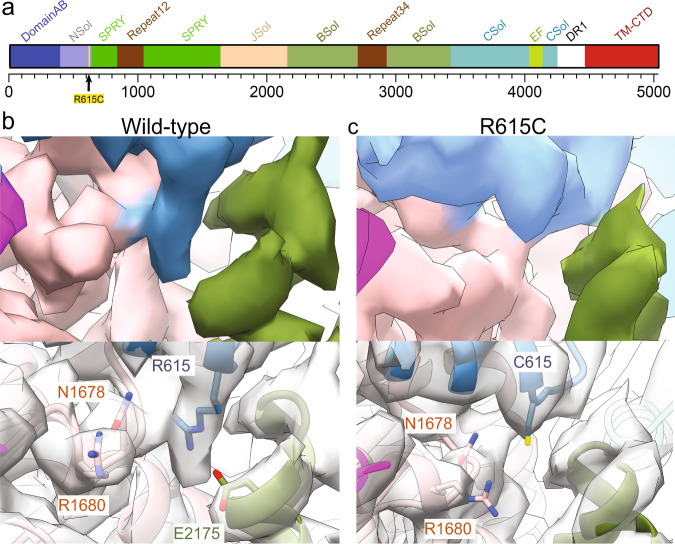
Arg615 mediates interactions between the Nsol, Bsol, and Jsol regions. **a** Schematic view of various structural elements in RyR1, using color coding used through the manuscript. The numbers below represent the amino acid residue numbering. The relative position of R615 is indicated. Nsol, N-terminal solenoid; SPRY, cluster of three SPRY domains; Jsol, Junctional solenoid; Bsol, Bridging solenoid; Csol, Central Solenoid; EF, EF-hand domain; DR1, Divergent region 1; TM-CTD, transmembrane region and C-terminal domain. **b** Map for WT pRyR1 (closed) highlighting the Arg615 sidechain. The Cryo-EM density is colored according to the scheme in panel (**a**). **c** Cryo-EM density of R615C pRyR1, showing a detail around residue 615. The WT map was resampled on the grid of the R615C pRyR1 map to allow for an unambiguous comparison. Volumes displayed for both maps are comparable (~710,000 A^3^) with a *σ* of 2.5. This shows clear loss of the Arg615 side-chain density in the mutant.

With the expected functionality of the R615C mutation confirmed, we solved cryo-EM structures of wild-type and R615C pRyR1, under nominally Ca^2+^-free conditions (5 mM EGTA), in the presence of FKBP12.6, which was used as a bait protein for purification (Supplementary Fig. 1). In both cases, one major 3D class was obtained, along with a smaller class with poor quality that was not further considered. Density modification or symmetry expansion and local masking of the major class drastically improved the local resolution throughout the structure (Supplementary Figs. 2–7, supplementary Table 1). Importantly, for WT pRyR1, we can observe unambiguous density for the Arg615 sidechain (Fig. 2). The location of this residue seems critical: Arg615 is situated in a junction between three solenoid domains: the N-terminal solenoid (Nsol, residues 395–630) containing Arg615, the junctional solenoid (JSol, residues 1657–2145), and the bridging solenoid (Bsol, residues 2146–3613). Nearby residues that form potential interactions with Arg615 are Asn1678 in Jsol and Glu2175 in Bsol (Supplementary Fig. 8).

**Fig. 3:**
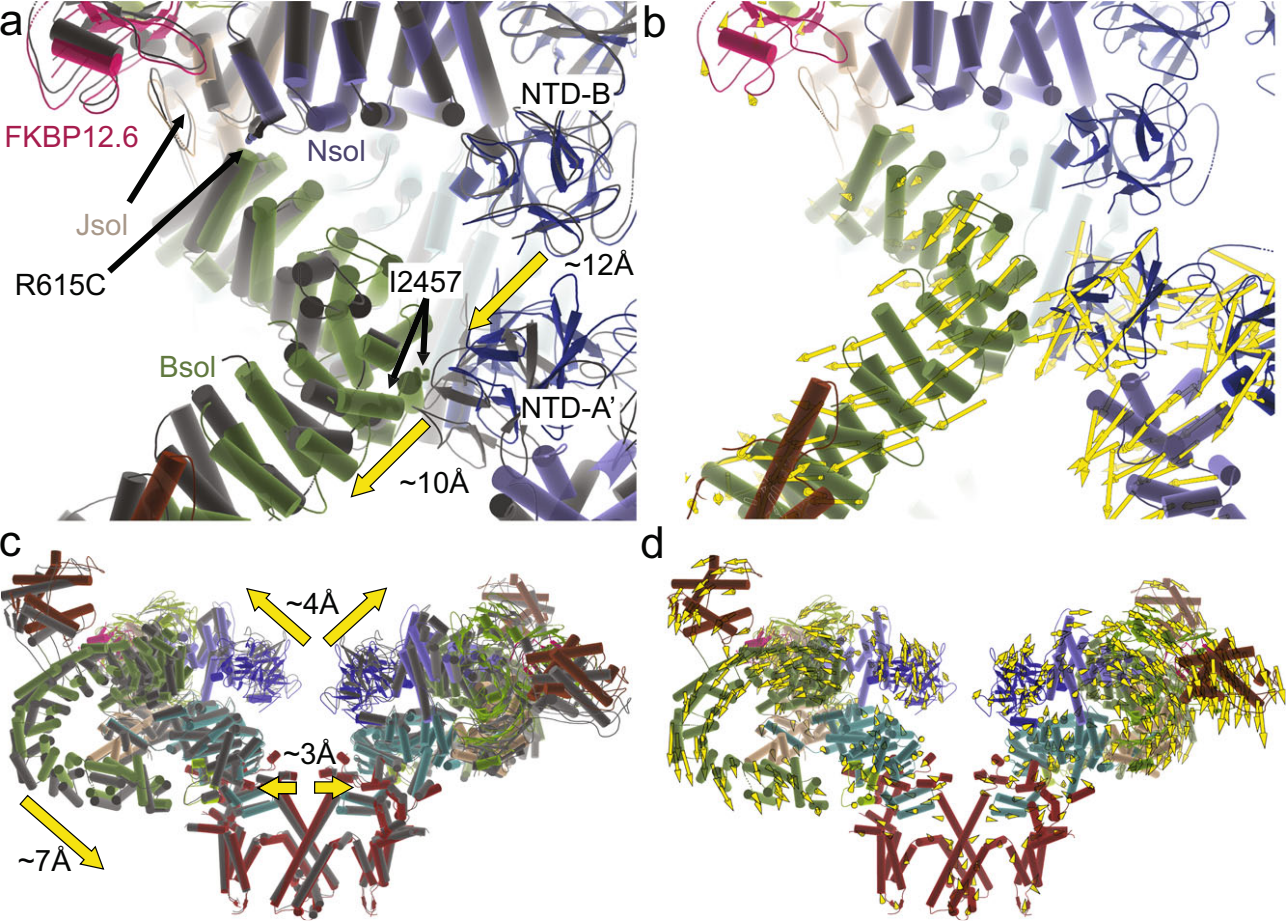
The R615C mutation induces opening via disruption of Nsol–Bsol interactions. **a** Superposition, based on the Nsol region, for WT (colors) and R615C (black) pig RyR1 (pRyR1). Alpha helices are represented as cylinders. This shows the movement of the Bsol relative to the Nsol, and the widening of the gap between the N-terminal domains of two neighboring subunits. The prime in NTD-A′ indicates that this is a different subunit. See also Movie S1. **b** Same view as in panel (**a**), showing the WT only, with yellow vectors showing the shifts >2 Å in the Bsol region compared to R615C pRyR1. **c** Side view of an overall superposition of WT (colors) and R615C (black) pRyR1. The major movements are indicated by arrows. Only two subunits are shown for clarity. See also Movie S2. **d** Same view as in panel (**c**), showing the WT pRyR1 only, with yellow vectors showing the shifts >2 Å throughout the protein going from WT to R615C pRyR1.

Despite identical experimental conditions, the overall R615C pRyR1 resolution is lower than for WT, potentially indicating a larger inherent heterogeneity. Density modification or symmetry expansion combined with local masking resulted in an improved resolution around the site of the mutation (Supplementary Figs. 3–7). A comparison with WT pRyR1 reveals the allosteric mechanism by which a mutation >130 Å away from the pore can facilitate its opening. Superposing the N-terminal solenoid (Nsol) shows that loss of interactions mediated by Arg615 leads to pivoting movements of the neighboring solenoid regions (Bsol, Jsol) relative to Nsol (Fig. 3a, b, Supplementary Fig. 7a, Movie S1). Locally, this results in shifts ~2–3 Å in the Bsol region near residue Glu2175, but due to the pivoting movement this is amplified further away from the mutation site, e.g., up to ~10 Å in the Bsol region around residue 2457 (Fig. 3a, b).

As the latter region of the Bsol makes contacts with the N-terminal domain A (NTD-A) of a neighboring subunit, the relative movement of the Nsol and Bsol regions results in substantial overall changes in RyR1. The N-terminal disease hot spot domains (NTD-A and NTD-B), located around the central 4-fold symmetry axis, are no longer forming inter-subunit interactions with the N-terminal domains of neighboring subunits, as observed for WT RyR1 (Fig. 3a, b, Movie S1 and S2). This result confirms previous predictions based on crystal structures of wild-type and disease mutant versions of the N-terminal disease hot spot^20,21^. The overall result is a widening of the cytosolic cap, with the corner regions moving downward toward the SR membrane (Fig. 3c, d, Movie S2). Corresponding movements in the Jsol region cause a splaying of the C-terminal region, which is directly linked to the S6 inner helices of the pore domain. As a result, the pore is considerably widened, consistent with an open channel based on the position of the S6 helices. The narrowest point in WT pRyR1, found at Ile4935, is now wide enough to support permeation (Supplementary Figs. 8c–10, Supplementary Table 2).

This is in contrast to our planar lipid bilayer experiments that indicate the channel should be closed under nominally Ca^2+^-free conditions. We hypothesize that this is because the channel was suspended in a detergent/lipid environment and not within a lipid bilayer. Indeed, CHAPS is known to affect channel opening intrinsically^25^. This is a general limitation of all RyR1 and RyR2 structures that have been solved in conditions with CHAPS detergent. However, given that both WT and mutant were prepared and solved under identical experimental conditions, this highlights the inherent effect of the mutation to facilitate channel opening. In this regard, the cryo-EM conditions can be considered as an activating condition. For wild-type pRyR1, the major class still represents closed channels, but for R615C pRyR1 the combination of cryo-EM condition and mutation results in mostly open channels. Thus, both the planar lipid bilayer assays and the cryo-EM structures confirm the intrinsic ability of the R615C mutation to facilitate channel opening.

How does the R615C mutation accomplish this? A direct comparison with available open and closed RyR1 structures readily reveals the mechanism of the disease mutation. In WT rabbit RyR1, the addition of Ca^2+^, ATP, and caffeine results in a proportion of open channel structures^11^. In this case also, the angle between the Nsol and Bsol increases upon channel opening (Supplementary Fig. 8a, b). Thus, interactions between Nsol and Bsol, in part mediated by Arg615, need to be disrupted for channel opening to occur. As the mutation intrinsically affects such interactions, weakening the Nsol–Bsol interface, less energy would be required to rearrange the Nsol and Bsol region, readily allowing the conformational change to occur at lower ligand concentrations.

Of note, the relative angle between the Nsol and Bsol region is larger for the R615C structure compared to the WT rabbit RyR1 structure activated with Ca^2+^, ATP, and caffeine (PDB 5TAL) (Supplementary Fig. 8b), and the mutation thus results in a distinct, pathological conformation. The Bsol region contains the location of the central disease hot spot, and clearly makes interactions with the N-terminal disease hot spot. Although the mutation results in a distinct conformation, we do not observe evidence of a disrupted interaction between the Bsol and the NTD-A, counter to the previous hypothesis that the interaction between the N-terminal and central disease hot spot may get disrupted or ‘unzipped’ in pathological conditions^15^.

In order to obtain a full picture of the effect of R615C, we aimed to compare the structures in both open and closed states. We therefore considered using Calmodulin, a well-known regulator of RyRs.

### Calmodulin has divergent effects on wild-type and disease mutant RyR1

**Fig. 4:**
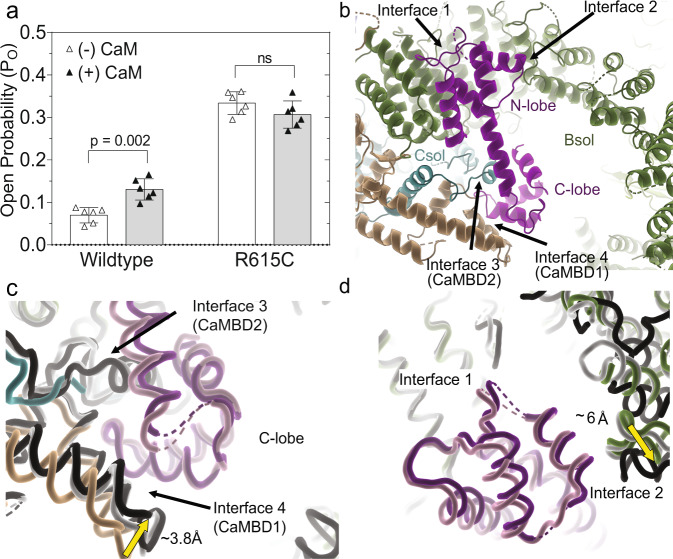
Effect of apoCaM on RyR1. **a** Single-channel open probability (P_o_) of wild type and R615C RyR1 with (filled) and without (open) 1 μM calmodulin (mean ± SD, Mann–Whitney two-tailed *t*-test, *n* = 6 with each independent recording corresponding to a newly incorporated channel from a different liposome) at low calcium ([Ca^2+]^_free_ = 40 nM) and with 2 mM ATP. Single-channel recordings were carried out under symmetrical conditions ([*cis*] = [*trans*]) with a constant −60 mV holding potential. **b** Binding of apoCaM (purple) at the periphery of the cytosolic cap, showing contacts of the N-lobe with two regions of the Bsol (interface 1 and interface 2). The C-lobe interacts with CaMBD1 and CaMBD2 segments. **c** Superposition, based on interface 1 helices, of WT pRyR1 without CaM (colors) on closed pRyR1+apoCaM (white) and open pRyR1+apoCaM (black). The corresponding apoCaM chains are shown in light (closed pRyR1) and dark purple (open pRyR1). The panel shows a detail around the C-lobe, showing the relative movement of the Jsol toward the C-lobe compared to the structure without apoCaM bound. CaMBD2 is only visible in the apoCaM bound structure. The interactions in this area are very similar for open and closed pRyR1 structures with apoCaM bound. **d** Same superposition as in panel (**c**), but showing the interfaces with the N-lobe. The interface 2 interaction is different for the open pRyR1 in complex with apoCaM.

Calmodulin (CaM) is a ubiquitous Ca^2+^ sensor that has been shown to bind and regulate RyR1. For RyR2, CaM has thus far only been shown to inhibit the channel^30^, but the effect on RyR1 is divergent, with CaM increasing the Po at low Ca^2+^ concentrations, while inhibiting it at high cytosolic Ca^2+^ ^31–34^. Dantrolene, a small molecule used clinically to treat MH, was previously found to only have an effect in the presence of CaM^35^, although this effect has been questioned^36^. We thus investigated the effect of CaM, at low (40 nM) Ca^2+^ concentrations, for WT and R615C pRyR1 in planar lipid bilayers. At such low Ca^2+^ concentrations, the intrinsic Po is too low to reliably detect an effect, but this can be circumvented by adding an activating ligand^32^. We therefore added 2 mM ATP to obtain a sufficiently high initial P_o_. Under these conditions, we find that the addition of CaM increased the P_o_ of wild-type pRyR1 ~2-fold, thus confirming the stimulatory role of CaM at low Ca^2+^, at least when another activating ligand is present. However, under the same conditions, CaM does not increase the P_o_ of R615C pig RyR1 and instead displayed a statistically insignificant decrease in P_o_ (Fig. 4a).

In order to understand how apoCaM engages with WT and mutant pRyR1, we solved cryo-EM structures of CaM compIexes at nominally Ca^2+^-free conditions, obtained by adding excess EGTA. For WT pRyR1, this resulted in two main classes that could both be refined and with structures built. The main class, corresponding to 70% of the particles, represents closed channels, whereas 27% of the particles contributed to an open channel class (Supplementary Fig. 11). Similarly, two classes and corresponding structures were obtained for R615C pRyR1 in the presence of apoCaM, with 69% open, and 19% closed channels (Supplementary Fig. 11). Importantly, the cryo-EM condition does not contain ATP, and the results have to be interpreted in the context of the cryo-EM condition. So, although the channels are normally closed in the presence of apoCaM and without any other activating molecules, in the cryo-EM condition we can see a significant fraction of open channels. Of note, despite identical conditions used for WT and R615C pRyR1 in the presence of apoCaM, there is still a larger proportion of open channels for R615C pRyR1, further highlighting the inherent effect of this mutation to facilitate channel opening.

As the bilayer shows that apoCaM stimulates opening of WT, but not R615C pRyR1, apoCaM seems to reduce, but not eliminate the relative differences between the two channels. Since the addition of apoCaM resulted in reliable reconstructions for both open and closed versions of WT and R615C pRyR1, this allowed us to compare the structures in different states, and to analyze the intrinsic effect of apoCaM on their conformations.

### Structural effect of calmodulin on WT pig RyR1

A more detailed analysis around the binding site of apoCaM reveals the nature of the two conformations. Starting with the structure of the closed complex, the N-lobe is the best resolved (Supplementary Figs. 12 and 13), and mostly makes contacts with α helices in the N-terminal part of the Bsol region, encoded by residues 2190–2242 (=interface 1) (Fig. 4b). In addition, the N-lobe is close to a short loop (~2595–2600) connecting two helices further downstream in the Bsol region (=interface 2). The C-lobe region is poorly resolved, but can be seen to make interactions with a helical region encoded by residues 3627–3634 (interface 3), previously found to interact with CaM in vitro and dubbed CaMBD2 (=CaM binding domain 2)^22,23,37^. Additional interactions include two helices in the Jsol region (=interface 4), including a peptide (residues 1975–1999) previously identified as CaMBD1^38,39^.

Comparing structures of WT pRyR1 with and without apoCaM, both in the closed state, it is clear that apoCaM introduces conformational changes: using interface 1 as a reference point, the Jsol region including interface 4 is moving closer to interact with the C-lobe (Fig. 4c). Despite these changes, which propagate into the whole cytosolic shell, there is no discernable effect on the channel pore.

**Fig. 5:**
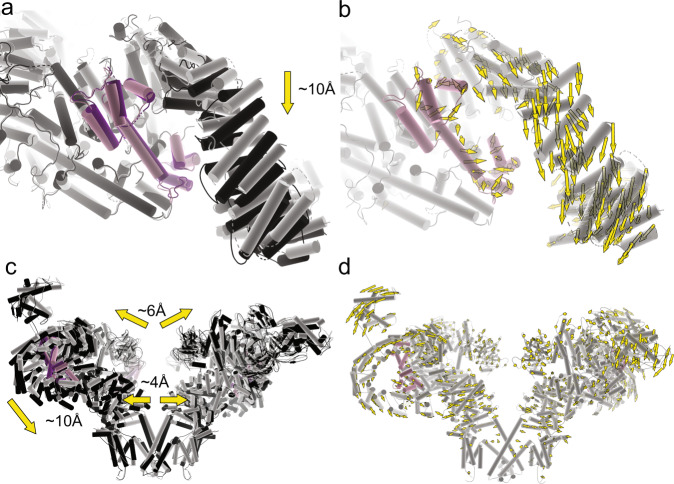
Structural affects of apoCaM on WT RyR1. **a** Superposition of closed (white + light pink) and open (black + dark purple) WT pRyR1 with apoCaM bound. The superposition is based on the interface 1 helices, showing the relative bending of the Bsol region. **b** Same view as in panel (**a**), showing the closed WT pRyR1+apoCaM structure and with vectors showing the positional shifts >2 Å in apoCaM and Bsol going from closed to open WT pRyR1+apoCaM. **c** Overall superposition of closed (white + light pink) and open (black + dark purple) WT pRyR1 with apoCaM bound. Side view with only two subunits shown for clarity. The main motions going from closed to open are indicated with arrows. **d** Same view as in panel (**c**), showing the closed WT pRyR1+apoCaM structure and with vectors showing the positional shifts >2 Å going from closed to open WT pRyR1+apoCaM.

In the open structure with apoCaM, interface 1 appears nearly identical to the closed structure with apoCaM bound. Using interface 1 as a reference point, there is no visible difference in the contacts with the C-lobe comparing open and closed structures with apoCaM bound (Fig. 4c, Movie S3). However, interface 2 with the N-lobe is altered, including shifts up to ~6 Å in the contacting region (Fig. 4d, Movie S3). This results in an overall bending of the Bsol region in the open versus closed structure, with positional shifts up to 10 Å in the Bsol region far from the apoCaM binding site (Fig. 5a, b). This has very large effects on RyR1: as the Bsol interacts with multiple regions, including the SPRY domains of a neighboring subunit, its bending results in motions within the Jsol, Csol, and ultimately the CTD and pore-forming region. There is an overall downward and outward motion of the cytosolic shell, and inter-subunit interactions in the N-terminal region are disrupted, similar to the movements seen for RyRs upon binding activating ligands^11^ (Fig. 5c, d). ApoCaM can thus promote channel opening by bending of the Bsol region, induced by the N-lobe.

Of note, a previous structure of pig RyR2 in complex with apoCaM (PDB 6JI8) has not shown this bending of the Bsol region^23^ (Supplementary Fig. 13). As the main regions that interact with apoCaM appear conserved between pRyR1 and pRyR2 (Supplementary Fig. 13), the structural differences may rather be explained by various sequence differences scattered throughout the Bsol region of pRyR1 and pRyR2, affecting the interactions allosterically.

### Structural effect of apo-calmodulin on R615C pig RyR1

The interfaces between apoCaM and pRyR1 are conserved between WT and R615C. The C-lobe forms interactions with CaMBD1 and CaMBD2, and these do not differ between the open and closed structures of R615C pRyR1:apoCaM. The N-lobe can interact with the Bsol region in two different modes, with the open state complex involving a bending of the Bsol region, induced by movement of interface 2, just like in the WT pRyR1:apoCaM complexes. Thus, at nominally zero Ca^2+^ levels, the interactions of apoCaM with R615C pRyR1 are nearly indistinguishable from apoCaM interacting with WT pRyR1.

**Fig. 6:**
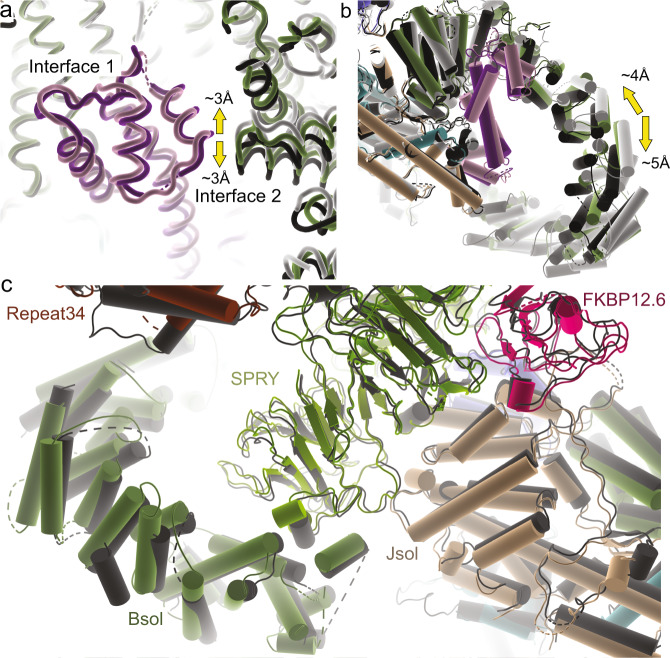
ApoCaM binding to R615C pRyR1 results in two conformations different from the structure without apoCaM. **a** Superposition, based on interface 1 helices, of R615C pRyR1 without apoCaM bound (colors) and with apoCaM bound in the closed (white) and open (black) conformation. The corresponding apoCaM chains are shown in light (closed) and dark (open) purple. The interface 2 region for R615C pRyR1 without apoCaM is in an intermediate conformation and has to move in one of two opposing directions upon apoCaM binding. **b** Same superposition as in panel (**a**), showing how the bending of the Bsol downstream of interface 2 is also intermediate for R615C pRyR1 without apoCaM bound. **c** Overall superposition of R615C pRyR1 (colors) and open R615C pRyR1+apoCaM (dark gray), showing that the conformational differences in the Bsol are transferred to the SPRY domains and Jsol of a neighboring subunit.

How then does the addition of CaM at low Ca^2+^ promote opening of WT, but not R615C pRyR1, as observed in the bilayer experiments? It is clear that there are two relatively stable conformations of the Bsol region and in the way the Bsol region interacts with the N-lobe at interface 2 (Fig. 6a, b; Movie S4). One of these two conformations, corresponding to a closed pRyR1, is already observed for WT pRyR1 in the absence of apoCaM. However, for R615C pRyR1, the main conformation of the Bsol region without apoCaM bound is different from either of the two conformations with apoCaM. Instead, it can be best described as a conformation that is ‘intermediate’ between the two when considering the degree of bending of the Bsol region. Thus, upon binding, apoCaM pushes the R615C pRyR1 into the same two conformations observed for WT pRyR1, with one corresponding to an open, and the other to a closed channel. Thus, the original major conformation of the Bsol in R615C pRyR1 without apoCaM bound no longer exists in the presence of apoCaM.

As the ‘intermediate’ conformation for the R615C pRyR1 without apoCaM corresponds to an open channel, the addition of apoCaM thus results in a redistribution of such open channels to a mixture of both open and closed channels. This would imply that apoCaM partially inhibits opening of R615C, but in the bilayer data (Fig. 4a) this effect is not statistically significant.

**Fig. 7:**
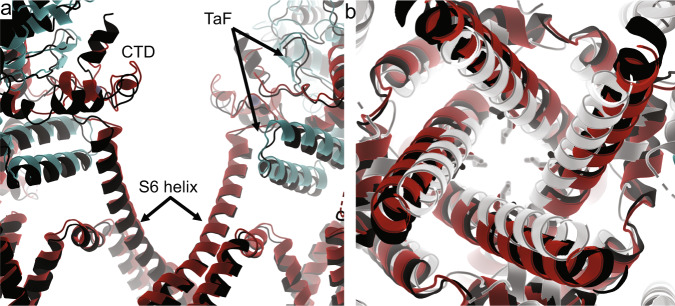
ApoCaM binding to R615C pRyR1 results in a mix of open and closed channels. **a** Overall superposition of R615C pRyR1(colors) and open R615C pRyR1+apoCaM (black), showing that, although both channels are in an open state, the CTD and the area contacting this (TaF = ‘Thumb and Forefinger’ domain) are in a different state. **b** Overall superposition of the inner pore of R615C pRyR1 (red) with closed R615C pRyR1+apoCaM (white) and open R615C pRyR1+apoCaM (black). The sidechain of Ile4935 is shown in sticks.

The distinct state of R615C pRyR1 without apoCaM, different from both open and closed R615C pRyR1:apoCaM, is also visible throughout the structure. The different degree of bending of the Bsol region affects its contacts, including the SPRY domains of a neighboring subunit (Fig. 6c). Through links with the Jsol and Csol regions, this results in changes in the position of the ‘Thumb and forefinger’ (TaF) domain, which interacts directly with the CTD (Fig. 7a). Despite this, the position of the inner helix S6 and the width of the pore are very similar for R615C pRyR1 and open R615C pRyR1:apoCaM (Fig. 7a, b). In conclusion, binding of apoCaM to R615C pRyR1 thus disrupts the overall original conformation induced by the mutation.

### Comparison of WT and R615C RyR1 in different states

**Fig. 8:**
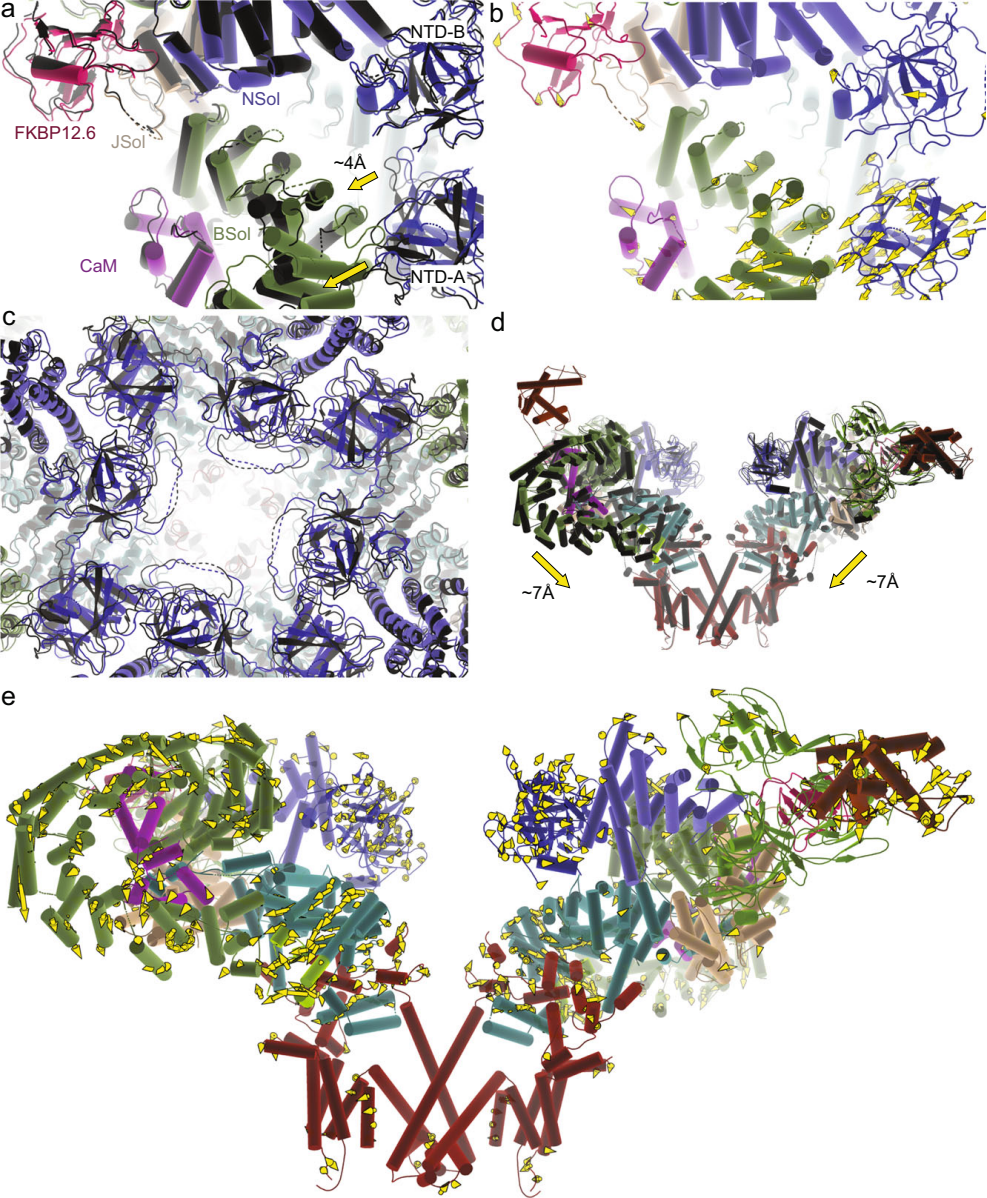
In the presence of apoCaM, the R615C mutation results in large changes when comparing the closed states of WT and mutant pRyR1. **a** Superposition, based on the Nsol, of closed WT pRyR1 + apoCaM (colors) and closed R615C pRyR1 + apoCaM dark gray), showing the relative movement of the Bsol and the NTD-A′ of a neighboring subunit. Despite apoCaM binding, the R615C pRyR1 still displays a pathological conformation. **b** Same view as in panel (**a**), but showing only the closed WT pRyR1 + apoCaM, with vector trajectories of alpha carbons with displacement >2 Å going from closed Wt pRyR1+apoCaM structure to closed R615C pRyR1+apoCaM. **c** Overall superposition of closed WT pRyR1+apoCaM (colors) and closed R615C pRyR1+apoCaM (dark gray), showing the changes in the NTD between different subunits. This is more readily visualized in Movie S5. **d** Same superposition as in panel (**c**), showing two subunits in a side view. This highlights the overall different conformational state of the closed WT pRyR1+apoCaM and closed R615C pRyR1+apoCaM. **e** Same view as in panel (**d**), but showing only the closed WT pRyR1+apoCaM, with vector trajectories indicating shifts >2 Å going from closed WT pRyR1+apoCaM to closed R615C pRyR1+apoCaM. The Repeat34 domain is not shown as it could not be placed reliably in the closed R615C pRyR1+apoCaM map.

Since the complexes with apoCaM resulted in open and closed state structures for both WT and R615C pRyR1, we can perform a more detailed analysis of the effect of the mutation within each state. Comparison of the closed WT and R615C pRyR1, both in the presence of apoCaM, shows that the mutation still results in conformational differences. There is a relative tilt between Nsol and Bsol, resulting in shifts ~2–7 Å across the entire Bsol region (Fig. 8a, b). As the Bsol interacts with the NTD-A of a neighboring subunit, this results in relative movements of the N-terminal domains (Fig. 8c), and downward movements of the cytosolic cap overall (Fig. 8d, e, Movie S5). Although the pore is closed, it is ~0.8 Å wider for closed R615C, as measured by the position of the Cα atom at Ile4935 (Movie S5, Supplementary Table 2). Comparing closed channels, the R615C mutation thus still results in a pathological conformation, with partial movements generally linked to channel opening. Indeed, the planar lipid bilayer shows that the P_o_ of R615C remains higher than WT even in the presence of apoCaM. ApoCaM can dampen, but not fully prevent a pathological conformation induced by the mutation, which remains more sensitive to activating ligands than WT.

**Fig. 9:**
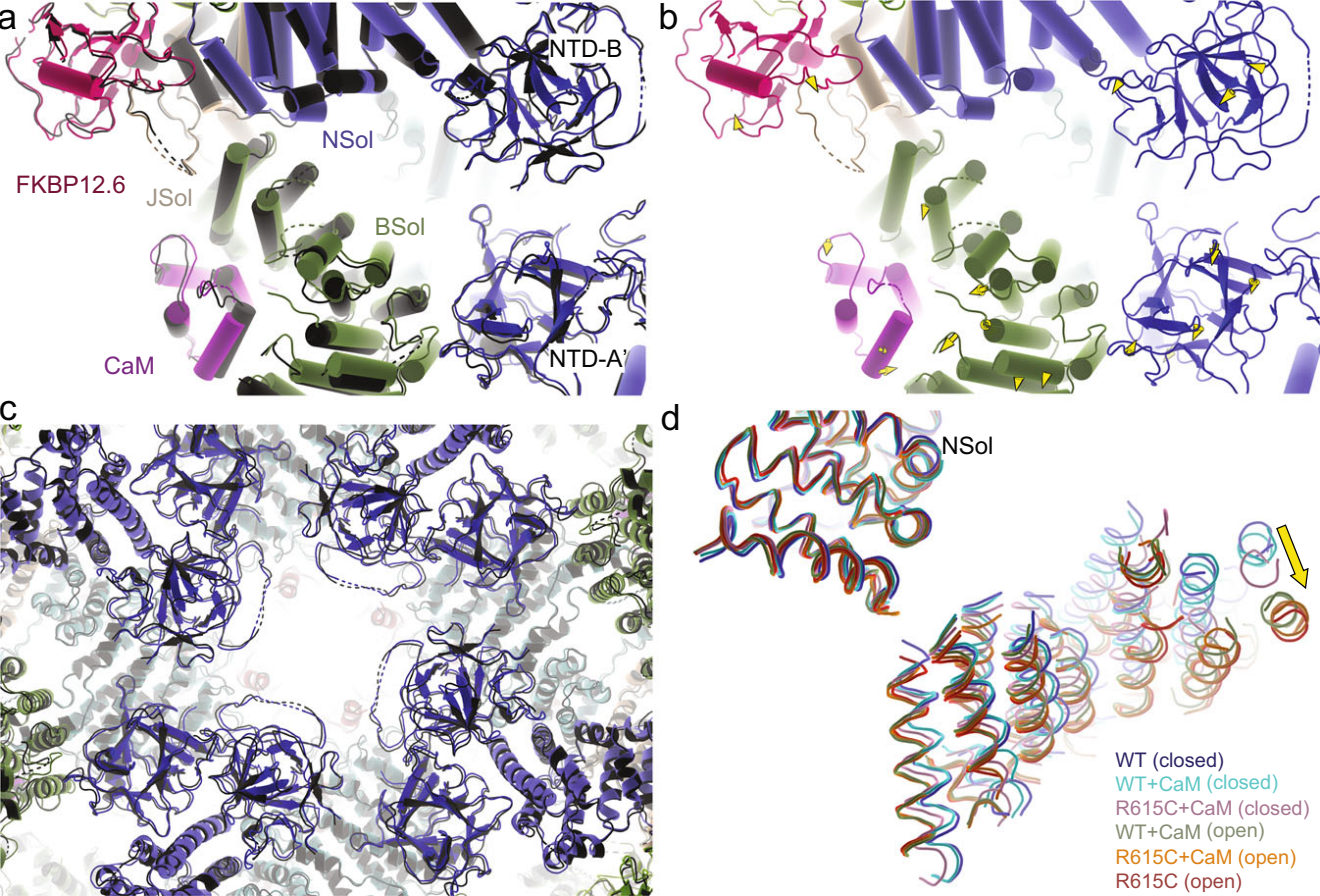
In the presence of apoCaM, the R615C mutation results in only subtle changes when comparing the open states of WT and mutant pRyR1. **a** Superposition, based on the Nsol, of open WT pRyR1+apoCaM (colors) and open R615C pRyR1+apoCaM (black). In this state, the differences between WT and R615C are subtle. **b** Same view as in panel (**a**), but showing only the closed WT pRyR1 + apoCaM, with arrows indicating the extent of shifts >2 Å going from open WT pRyR1+apoCaM to open R615C pRyR1 + apoCaM. The extent of the shifts is much smaller in relation to the closed structures (Fig. 8b). **c** Overall superposition of open WT pRyR1+apoCaM (colors) and open R615C pRyR1+apoCaM (black), with a close-up around the NTDs of different subunits, highlights the similarity between WT and R615C in this state. **d** Superposition, based on the Nsol, of all six structures described in this study. This highlights the range in the angle of the Bsol relative to the Nsol, in the order WT (no CaM) ≈ closed WT + apoCaM < closed R615C + apoCaM < open WT + apoCaM < open R615C + apoCaM ≈ R615C (no CaM).

Comparing the open structures in complex with apoCaM, the differences between WT and R615C pRyR1 are much smaller. Relative to the Nsol, the movements in the Bsol are dampened to 1 Å around residue 2443 (Fig. 9a, b). Similarly, the relative positions of the inner helices within the pore are almost identical, and there is little additional movement in the N-terminal region (Fig. 9c; Movie S6). Figure 9d compares the relative tilt of the Bsol region across all structures presented in this study, showing the relative tilt angle in the following increasing order: WT (no CaM) = closed WT + apoCaM < closed R615C + apoCaM < open WT + apoCaM < open R615C + apoCaM = R615C (no CaM). Since tilting of Bsol relative to Nsol is generally involved with channel opening, its interface, close to the hinge point, presents an attractive allosteric pocket for novel molecules with therapeutic potential.

### Species-specific differences

As previous cryo-EM studies have focused on rabbit RyR1, we compared potential structural differences between rabbit and pig RyR1 (Supplementary Fig. 14). Not surprisingly, the structures align very well, with the differences only found in regions that are generally more flexible. This includes the Repeat34 domain, which is poorly resolved in all RyR cryo-EM reconstructions. Minor differences were also observed for the N-terminal domains (2–4 Å r.m.s.d.), SPRY domains (3–5 Å r.m.s.d.), and repeat 12 domain (3–6 Å r.m.s.d.) (Supplementary Fig. 14).

## Discussion

MH is a potentially lethal condition whereby patients with mutations in RyR1 or other proteins are particularly sensitive to volatile halogenated anesthetics or muscle relaxants such as succinylcholine. Hundreds of mutations in RyR1 have been linked to the disorder, but only a handful have been investigated functionally. Within those, a common theme is that the mutations facilitate channel opening, leading to enhanced sensitivity to channel activators. The R615C mutation in pRyR1 is the founder mutation that has linked RyRs to a range of debilitating disorders. In order to gain insights into the disease mechanism, we set out to solve structures of WT and mutant pRyR1 in both open and closed states.

Our study shows that the mutation has a dual effect on the RyR: it results in distinct pathological conformations, and it facilitates channel opening by interfering with salt bridge interactions between the Nsol and Bsol regions. The latter is explained by its location at a domain–domain interface: sandwiched between three solenoid regions, it weakens their interactions, thus facilitating their relative movement during channel gating. As many disease mutations in both RyR1 and RyR2 are found at domain–domain interfaces^3^, we postulate that many act via a similar mechanism. Supplementary Fig. 15 gives an overview of various disease mutations found in RyR1. Indeed, using the list of diagnostic RyR1 mutations, as defined by the European Malignant Hyperthermia Group (emhg.org), already 6 mutations are near the Nsol–Bsol interface, but many more have sequence variants, for which pathogenicity remains to be established, can be found in the same vicinity (https://gnomad.broadinstitute.org/).

An important point to keep in mind is that cryo-EM analysis of RyRs typically involves detergents (CHAPS, Tween-20), which interfere with function. Their ability to affect the conformation of the channel is immediately evident from cryo-EM structures of RyR1 in the presence of PCB-95^26^. When using Tween-20 as a detergent, the structure showed closed channels, but in the presence of CHAPS the channels were open, showing how the precise detergent strongly affects opening. Membrane mimetics such as lipid nanodiscs have been proposed as good alternatives to detergents, but unfortunately this does not solve the problem. When reconstituted in lipid nanodiscs, the RyR1 particles adopt a preferred orientation that prevents reliable 3D reconstructions^25^. This can be circumvented by the use of non-solubilizing fluorinated detergents, but unfortunately these also affect the intrinsic gating of RyRs^25^. Thus, when considering the effect of additional proteins and small molecules on channel gating, only relative comparisons can be made. Whereas the structures can provide mechanistic explanations for an effect, the effect itself needs to be observed in the context of a lipid membrane bilayer. However, one expectation is that the addition of effectors should follow the same trend in both the conditions used for cryo-EM and within the bilayer.

Our results suggest that the cryo-EM conditions we used intrinsically facilitate channel opening: at low Ca^2+^ and without any additional effectors, R615C pRyR1 should be closed but we find instead open channels. Once this enhancing effect of the cryo-EM conditions is taken into consideration, the observations we made do follow the same trend as in the bilayer. At low Ca^2+^, and without any other effector molecule, the Po of RyR1 is very low for both WT and R615C pRyR1. However, when adding ATP, a known enhancer, it is clear that the Po increases for both, but that the R615C mutation has a much higher Po than WT (Fig. 4a). In the cryo-EM condition, which lacks ATP, this trend is reflected by the fact that the WT structure predominantly showed closed, and the R615C structure predominantly showed open channels. Similarly, in the presence of ATP and apoCaM, the Po of R615C pRyR1 is significantly higher than for WT pRyR1. Correspondingly, in the cryo-EM condition, the WT pRyR1 + apoCaM dataset shows mostly closed channels, whereas the mutant shows mostly open channels.

The bilayer experiments show that apoCaM stimulates opening of WT pRyR1 (at least when an activator like ATP is present). In contrast, under the same conditions it has no statistically significant effect on opening of R615C pRyR1 (Fig. 4a). Thus, apoCaM does not reduce Po of R615C pRyR1, but seems to reduce the relative difference in Po between WT and R615C pRyR1. As the affinity for full-length RyR1 has been estimated to be ~30 nM Kd at low Ca^2+^ levels^40^, and we utilized 1 μM CaM in the bilayer experiments, the binding sites should be saturated. 2D classification results of the datasets in presence of apoCaM also did not reveal clear evidence of apoCaM sub-saturation, when compared to simulated 2D projections of WT RyR1 without and with apoCaM (Supplementary Fig. 16a–d). However, it will be important to measure the affinity of apoCaM for R615C pRyR1, as a reduced affinity may have implications when the free CaM concentration in the cytosol is not sufficient to saturate all binding sites in physiological conditions.

Our cryo-EM data show that apoCaM binding also has different structural effects on WT and mutant pRyR1, as its binding is not compatible with the original pathological conformation of R615C pRyR1 without apoCaM bound. This study thus highlights the importance of considering additional auxiliary proteins and ligands, which may affect the interpretation in both functional and structural experiments. Therefore, it will be important to further investigate the complex interplay between the disease mutation and the many other known RyR regulators, including the Ca^2+^-bound form of CaM.

The study also highlights the role of the bridging solenoid (Bsol) as a key element in transmitting the effects of a disease-causing mutation, but also of protein modulators like CaM. The relative tilt and degree of bending Bsol can dictate the open state as evidenced by the significant impact of apoCaM on the conformations of pRyR1. The pocket formed at the junction of the Nsol and Bsol region is thus an attractive candidate for small molecules that could stabilize the closed state where the tilt is minimal. Such a molecule is predicted to be an allosteric antagonist. The Bsol also forms the location for the central disease hot spot of RyRs.

These structures provide key insights into the pathophysiology of RyR, as a detailed comparison of open and closed states for any disease mutation has not been reported before. As MH is not just caused by a mutation, but also needs triggering ligands such as volatile anesthetics, it will be of great interest to identify their binding site and effect on conformation.

During revision of this manuscript, a study was published looking at the effect of a different disease mutation (R164C) in rabbit RyR1 and its ortholog^41^. In this case, however, the effect of CaM was not considered, and the mutation yielded more subtle changes that did not trigger channel opening (Supplementary Fig 11), suggesting the R164C mutation is milder. Future studies including the open state of this mutant, in the presence of other modulators like CaM, will be required to provide insights into the similarities and dissimilarities of distinct disease mutations.

## Methods

### Expression and purification of GST-FKBP12.6 and CaM

Human FKBP12.6 was cloned into a modified pGEX vector with an N-terminal 6× His-tag, a GST tag, and a TEV cleavage site. Calmodulin (CaM) was cloned into a modified pET-28 vector containing a 6xHis-tag, MBP-fusion protein, and TEV cleavage site at the N-terminus. Both proteins were expressed using *E. coli* Rosetta^TM^ (DE3) pLysS strain (Novagen). The cells were grown at 37 °C in 2YT medium until OD_600_ reached ~0.6–0.8, and then were induced with 0.4 mM β-D-1-thiogalactopyranoside (IPTG) and grown at 37 °C for another 6–8 h. Cells were harvested by centrifugation at 8000 × *g* for 15 min at 4 °C. Cells expressing GST-FKBP12.6 were resuspended and lysed via sonication in lysis buffer (10 mM HEPES, pH 7.4, 250 mM KCl, 25 μg/mL DNase I, 25 μg/mL lysozyme, 10 mM imidazole, and 1 mM phenylmethanesulfonyl fluoride (PMSF)). The lysate was centrifuged at 40,000 × *g* for 30 min at 4 °C and the supernatant was filtered through a 0.22-μm filter. The sample was loaded onto 10 mL of pre-equilibrated immobilized metal affinity column (HisTRAP^TM^ MC, GE Healthcare Lifesciences), washed with 10 column volumes (CV) buffer A (10 mM HEPES, 250 mM KCl, pH 7.4) containing 10 mM imidazole and eluted with 5CV buffer B (10 mM HEPES, 250 mM KCl, 500 mM imidazole, pH 7.4). After dialyzing 3–4 h against a buffer containing 10 mM Tris–HCl, pH 8.8, precipitate was pelleted by centrifugation and the soluble protein was purified by an anion exchange column (HQ, GE Healthcare Lifesciences) with a linear gradient of 0–80% buffer C containing 10 mM Tris–HCl (pH 8.8), and 1 M KCl. The FKBP12.6-containing fractions were concentrated and injected on a gel-filtration column (Superdex 200, GE Healthcare Lifesciences) and eluted with buffer A.

Cell pellets expressing CaM were lysed in the same way, with the exception that the lysis buffer was supplemented with 10 mM CaCl_2_. The lysate was centrifuged at 40,000 × *g* for 30 min at 4 °C and the supernatant was filtered through a 0.22-μm filter. The sample was loaded onto 10 mL of pre-equilibrated immobilized metal affinity column (HisTRAP^TM^, GE Healthcare Lifesciences), washed with 10CV buffer A containing 10 mM CaCl_2_ and eluted with buffer B containing 10 mM CaCl_2_. The protein was dialyzed against 10 mM KCl, 10 mM HEPES, pH 7.4, and 10 mM CaCl_2_ overnight while simultaneously cleaved with TEV protease at 4 °C. The sample was applied to a 25-mL amylose column (New England Biolabs) and the flow-through applied to anion exchange column (HQ, GE Healthcare Lifesciences) with a linear gradient of 0–45% buffer C containing 10 mM CaCl_2_. Residual His-MBP-tag was removed by passage of the purified material through 5 mL of pre-equilibrated immobilized metal affinity column (HisTRAP^TM^, GE Healthcare Lifesciences) and the flow-through applied to a gel-filtration column (HiLoad 16/60 Superdex 75 prep grade, GE Healthcare Lifesciences) and eluted with buffer A containing 10 mM CaCl_2_. Finally, the sample was excessively dialyzed overnight at 4 °C against buffer A containing 10 mM EGTA. The peak fractions were collected and stored at −80 °C.

### Purification of WT and R615C pRyR1 from porcine muscle

WT porcine muscle was obtained from Pel-Freeze Biologicals. R615C porcine muscle was obtained from Boyle Farms (Moorehead, Iowa), where the R615C mutation was confirmed through high-resolution melt analysis by GenAlysis (Evergreen, Colorado). Upon arrival of the muscle tissue in the lab, both wild-type and R615C muscle tissue was sent to Neogen (Lincoln, Nebraska) for further analysis through the Sequenom MassARRAY platform^42^, which confirmed the latter tissue to be homozygous for the R615C mutation, which was absent from WT tissue.

Between 95 and 100 g of frozen porcine skeletal muscle was blended for 120 s in 500 mL of 15 mM Tris/maleate (pH 6.8), 75 mM NaCl, 1 mM DTT, 1 mM EDTA, 150 μM PMSF, and 1 mM benzamidine. The mixture was centrifuged at 4 °C for 10 min at 7000 × *g*. The supernatant was filtered through a cheesecloth and centrifuged at 4 °C for 35 min at 40,000 × *g*. Each pellet was solubilized in 50 mL of 20 mM Tris (pH 7.5), 1 M NaCl, 2 mM TCEP, and 2 mM EGTA, 1% CHAPS, and 0.1% soybean phosphatidylcholine with protease inhibitor cocktail (Protease Inhibitor Cocktail Set III, EDTA-Free, Calbiochem). The solubilized membranes were diluted with an equal volume of solubilization buffer without NaCl and centrifuged for 70 min at 110,000 × *g*. The supernatant was filtered using a 0.45 μm filter and 5 mg of purified His-GST-FKBP12.6 was added. We chose FKBP12.6 instead of FKBP12, as the former binds with higher affinity, resulting in higher yields. The solution was then applied to 2 mL Glutathione Sepharose 4B GST-tagged protein purification resin (GE Healthcare) at 4 °C. The resin was washed with 25 mM Tris (pH 7.5), 0.5 M NaCl, 2 mM TCEP, 2 mM EGTA, 0.2% soybean phosphatidylcholine and 0.5% CHAPS. Bound RyR1 was collected from the resin by either elution with glutathione or cleavage of the His-GST tag from FKBP12.6 with TEV protease. For elution with glutathione, 75 mM Tris (pH 8.0), 0.5 M NaCl, 2 mM TCEP, 1 mM EGTA, 0.2% soybean phosphatidylcholine, 0.5% CHAPS, and 10 mM glutathione was used. For TEV cleavage, the resin was incubated in 25 mM Tris (pH 7.5), 0.5 M NaCl, 2 mM TCEP, 2 mM EGTA, 0.2% soybean phosphatidylcholine, and 0.5% CHAPS containing 0.5 mg TEV for 16 h at 4 °C. An additional 0.5 mg TEV was then added and incubated for 3 h before the sample was eluted from the resin. The elution was concentrated to ~500 μL using 100 kDa cutoff concentrator (Millipore) prior to being loaded to a size exclusion column (Superose 6 30/100, GE Healthcare Lifesciences) and washed with 25 mM Tris (pH 7.5), 250 mM NaCl, 1 mM TCEP, 5 mM EGTA, 0.5% CHAPS and 0.001% DOPC. Peak fractions containing RyR1 complexes were combined and concentrated to ~0.2–5 μM. Concentration was estimated using NanoDrop (Abs at 280 nm, 1% (w/v) = 1.0). Preparations were used for either electrophysiology studies or electron microscopy.

### Preparation of RyR1 proteoliposomes

Purified RyR1 was reconstituted into proteoliposomes as described by Lee et al.^43^. Briefly, sample containing the pure complex was added to 2 mg/mL of lipid mixture (5:3 DOPE:DOPC, mol/mol). To form proteoliposomes the sample was dialyzed overnight using a 1 or 3.5 kDa membrane in 0.5 M NaCl, 0.1 mM EGTA, 0.2 mM CaCl_2_, 150 μM PMSF, 1 mM DTT, and 10 mM HEPES (pH 7.4). Following dialysis, the samples were flash-frozen with liquid nitrogen and stored at −80 °C.

### Single-channel recordings

For all experiments, planar lipid bilayers were formed from a mixture of DOPE:DOPC = 5:3 (mol/mol) dissolved in decane with a final lipid concentration of 15–20 mg/mL. For single-channel recordings, an integrated chip-based recording setup Orbit mini and EDR2 software (Nanion Technologies) was employed. Recordings were obtained in parallel with multielectrode-cavity-array chips (Ionera Technologies). The *cis* and *trans* chambers contained symmetrical solutions of 250 mM HEPES, 150 mM KCl, 1 mM EGTA (pH 7.3), 2 mM TCEP, and 1 mM CaCl_2_ ([Ca^2+^]_free_ = 0.1 μM–1 mM) with or without 2 mM ATP and 1 μM calmodulin. The latter is expected to saturate the RyRs given the estimated Kd of ~30 nM^40^. The free concentrations for Ca^2+^ were calculated with Ca-EGTA Calculator TS v1.3^44^ and verified using a PerfectIon™ Combination Calcium Electrode unit (Mettler Toledo). To promote fusion to prepared suspended bilayers, 5–15% glycerol was incorporated into the proteoliposomes. In brief, prepared proteoliposomes were mixed 1:1 in 20 mM HEPES (pH 7.3), 150 mM KCl, and 10–30% glycerol. The samples were then freeze-thawed and sonicated 3–5 times to incorporate the glycerol into the proteoliposome lumen. RyR1 proteoliposomes were added to the *cis* chamber. To further promote fusion the voltage was maintained at +40 mV. All RyR1 measurements were conducted at 22 °C and at a constant voltage of −60 mV. Recordings were filtered at a final bandwidth of 10,000 Hz. Clampfit (v10.6/v10.2) was used to analyze current traces and generate figures. Only channels with a conductance >700 pS were included in the analysis^45^.

### Electron microscopy

Purified WT and R615C pRyR1 particles were monodispersed as assessed by negative stain EM. Four microliter of purified RyR1 was applied to glow-discharged 400-mesh copper grids (Electron Microscopy Sciences) and stained with 0.7% (w/v%) uranyl formate. Negatively stained EM grids were imaged on a Tecnai G2 Spirit microscope (FEI) operated at 120 kV.

For cryo-EM on samples without apoCaM added, 3 μL of 0.4 μM purified RyR1 was placed on non-glow-discharged holey carbon quantifoil grids with a continuous 2 nm graphene oxide film. For cryo-EM with apoCaM, 4 μM RyR1 was used instead, with 20–125 μM apoCaM added to saturate the binding sites. The latter samples were applied to quantifoil grids without a graphene oxide support layer. Grids were blotted for 1–1.5 s and flash-frozen with liquid ethane using an FEI Vitrobot IV. Grids were transferred to an FEI Titan Krios electron microscope operating at 300 kV. Datasets were collected using the automated collection system EPU (Thermo Fisher, v2.6.0.59REL) at a nominal magnification of ×59,000 or ×75,000 in counting mode on a Falcon III detector calibrated to pixel size of 1.4 and 1.09 Å, respectively, using a gold cross grating. For collections at 1.4 Å/pixel a total dose of 30e−/Å^2^ was applied over 60 s and 24 frames. The first 22 frames were exposed to 1.2e−/Å^2^ per frame and the final 2 frames were exposed to 1.8e−/Å^2^ per frame. For collections at 1.09 Å/pixel a total dose of 50e−/Å^2^ was applied equally over 60 s and 48 frames.

### Image processing

Two image processing strategies were employed using cryoSPARC (v2.9/2.12.4)^46^ and/or RELION 3.0^47^. First, for datasets without apoCaM, which were collected using graphene oxide grids, CryoSPARC (v2.9/2.12.4) was used, including GCTF^48^ with equi-phase averaging for CTF correction. Particles were picked manually from each dataset to form templates for autopicking. Low-quality particles and contaminants were removed during 2D classification. 3D classification indicated 2 classes, one of which was of poor quality, which mostly contained particles in a preferred orientation. Particles in the better class underwent CTF refinement and homogenous refinement within cryoSPARC. Second, for datasets containing apoCaM, which were collected using holey carbon grids, the best results were obtained by using a strategy that employed both CryoSPARC (v2.9/2.12.4) and RELION 3.0. MotionCor2^49^ was used for patch motion correction followed by CTF correction using GCTF (v1.05)^48^ with equi-phase averaging. CrYOLO (v1.3.6)^50^ was used for particle picking. To remove low-quality particles and contaminants, picked particles underwent 2D classification within CryoSPARC and 3D classification within RELION. For these datasets with apoCaM present, we did not observe any 3D classes that indicated sub-saturation, as expected from using concentrations that are several orders of magnitude above the Kd. Final particles underwent refinement (C4 symmetry), CTF refinement and polishing in RELION 3.0. The final particle stacks were then evaluated for global heterogeneity with RELION 3D classification (C4 symmetry) using 3–5 classes with finer (1.8°) angular sampling. The final number of classes was determined by their reproducibility and the quality of the following class refinements. Stable unique classes underwent Resolve density modification as implemented in Phenix RESOLVE(v.dev-3714)^51^ using half-maps from RELION 3DAutoRefine resulting in considerable improvements to the densities within the core of RyR1 maps. In most cases, the maps improved by ~0.1–0.2 Å resolution based on FSC cutoff of 0.5 which is also reported by Terwilliger et al.^52^. The biggest improvement (0.2 Å using FSC = 0.143 or 0.9 Å using FSC = 0.5) was observed for R615C (open) dataset, which correlated with significant visible improvements observed between the original and density-modified maps (Supplementary Fig. 7).

As a separate means to improve the resolution of the datasets containing apoCaM, we also used focused refinement within RELION using C4 symmetry expanded particle sets. For the datasets without apoCaM, focused refinement in cryoSPARC did not yield satisfactory results. We therefore also employed the same pipeline as used for the datasets containing apoCaM, which allowed for focused refinement in RELION that improved the maps. Composite maps were generated in UCSF Chimera^53^ by fitting focused refined maps within the corresponding global maps. All data collection statistics are shown in Supplementary Table 1.

### Model building

Two models, R615C RyR1 + apoCaM (class 1) and wild-type RyR1 + apoCaM (class 1 & 2), which reflect open and closed channels respectively, were built using *Coot*^54^ with deposited PDB 5TAW as starting template. The individual lobes of apoCaM (PDB ID 6JI8) were placed by rigid-body fitting and manually adjusted in *Coot*. Models underwent iterative manual remodeling in *Coot* using density-modified and composite focused refinement maps generated in UCSF Chimera. Phenix (v.dev-3714, v.1.18.2) *real_space_refine* was applied to refine the full structure using the density-modified maps. The open and closed RyR1 models were then used as templates for iterative manual remodeling and refinement of the remaining models. Regions of low resolution within composite and/or density-modified maps, the phosphorylation domain (residues 2735–2940) and unassigned densities of the alpha-solenoid domain, were rigid-body fitted with the phosphorylation domain modeled from wild-type RyR1 + apoCaM (class 1 & 2) and poly-alanine helices (UNK), respectively. The final model validation statistics were calculated using Phenix (Supplementary Table 1).

### Model analysis

Animations and morphs between different structures were prepared using PyMOL 2.3 (Schrodinger). Local superpositions are based on selections of the indicated regions in PyMOL, whereas global superpositions of full-length RyR structures were generated by fitting all structures into the same map, EMD-22015, using UCSF Chimera. Atomic distances were measured using PyMOL 2.3 and UCSF Chimera. Pore radius calculations were generated using HOLE^55^ on the core region of the final atomic models (3680-5035) with default settings. FSC curves were plotted using GraphPad Prism 8.0.

### Statistical information

GraphPad Prism 8.0, unless otherwise noted, was used for statistical data analysis. Figures and graphs were generated using PyMOL, UCSF Chimera, and Prism 8.0 unless otherwise noted.

### Reporting summary

Further information on research design is available in the Nature Research Reporting Summary linked to this article.

## Supplementary information

Supplementary Information

Supplementary Movie 1

Supplementary Movie 2

Supplementary Movie 3

Supplementary Movie 4

Supplementary Movie 5

Supplementary Movie 6

Description of Additional Supplementary files

Reporting Summary

## Data Availability

Raw cryo-EM and planar lipid bilayer data that was used in support of the findings are available from the corresponding author upon reasonable request. The authors declare that all other data supporting the findings of this study are available within the paper and its supplementary information files. The structures are available via the Protein Data Bank (PDB) with accession codes: 6W1N (Pig Ryanodine Receptor (WT) in 5 mM EGTA condition), 6X32 (Wt pig RyR1 in complex with apoCaM, EGTA condition (class 1 and 2, closed)), 6X33 (Wt pig RyR1 in complex with apoCaM, EGTA condition (class 3, open)), 6X34 (Pig R615C RyR1 EGTA (all classes, open)), 6X35 (Pig R615C RyR1 in complex with CaM, EGTA (class 1, open)), and 6X36 (Pig R615C RyR1 in complex with CaM, EGTA (class 3, closed)), and via the Electron Microscopy Data Bank (EMDB) with entry number EMD-21513 (Wt pig RyR1, EGTA condition),  EMD-22015 (Wt pig RyR1 in complex with apoCaM, EGTA condition (class 1 and 2, closed)), EMD-22016 (Wt pig RyR1 in complex with apoCaM, EGTA condition (class 3, open)), EMD-22017 (Pig R615C RyR1 EGTA (all classes, open)), EMD-22018 (Pig R615C RyR1 in complex with CaM, EGTA (class 1, open)) and EMD-22019 (Pig R615C RyR1 in complex with CaM, EGTA (class 3, closed)). Source data are provided with this paper.

## Source data

Source Data

## References

[1] Van Petegem F (2012). Ryanodine receptors: structure and function. J. Biol. Chem..

[2] Priori SG (2001). Mutations in the cardiac ryanodine receptor gene (hRyR2) underlie catecholaminergic polymorphic ventricular tachycardia. Circulation.

[3] Pancaroglu R, Van Petegem F (2018). Calcium channelopathies: structural insights into disorders of the muscle excitation-contraction complex. Annu Rev. Genet..

[4] MacLennan DH, Phillips MS (1992). Malignant hyperthermia. Science.

[5] Quane KA (1993). Mutations in the ryanodine receptor gene in central core disease and malignant hyperthermia. Nat. Genet..

[6] Zhang Y (1993). A mutation in the human ryanodine receptor gene associated with central core disease. Nat. Genet..

[7] Efremov RG, Leitner A, Aebersold R, Raunser S (2015). Architecture and conformational switch mechanism of the ryanodine receptor. Nature.

[8] Yan Z (2015). Structure of the rabbit ryanodine receptor RyR1 at near-atomic resolution. Nature.

[9] Zalk R (2015). Structure of a mammalian ryanodine receptor. Nature.

[10] Yuchi Z, Van Petegem F (2016). Ryanodine receptors under the magnifying lens: insights and limitations of cryo-electron microscopy and X-ray crystallography studies. Cell Calcium.

[11] des Georges A (2016). Structural basis for gating and activation of RyR1. Cell.

[12] Ma R (2020). Structural basis for diamide modulation of ryanodine receptor. Nat. Chem. Biol..

[13] Kobayashi S (2005). Dantrolene stabilizes domain interactions within the ryanodine receptor. J. Biol. Chem..

[14] Bers DM (2002). Cardiac excitation-contraction coupling. Nature.

[15] Ikemoto N, Yamamoto T (2002). Regulation of calcium release by interdomain interaction within ryanodine receptors. Front. Biosci..

[16] Tateishi H (2009). Defective domain-domain interactions within the ryanodine receptor as a critical cause of diastolic Ca^2+^ leak in failing hearts. Cardiovasc Res..

[17] Lau K, Van Petegem F (2014). Crystal structures of wild type and disease mutant forms of the ryanodine receptor SPRY2 domain. Nat. Commun..

[18] Yuchi Z (2015). Crystal structures of ryanodine receptor SPRY1 and tandem-repeat domains reveal a critical FKBP12 binding determinant. Nat. Commun..

[19] Amador FJ (2013). Type 2 ryanodine receptor domain A contains a unique and dynamic alpha-helix that transitions to a beta-strand in a mutant linked with a heritable cardiomyopathy. J. Mol. Biol..

[20] Kimlicka L, Lau K, Tung CC, Van Petegem F (2013). Disease mutations in the ryanodine receptor N-terminal region couple to a mobile intersubunit interface. Nat. Commun..

[21] Kimlicka L (2013). The cardiac ryanodine receptor N-terminal region contains an anion binding site that is targeted by disease mutations. Structure.

[22] Lobo PA, Kimlicka L, Tung CC, Van Petegem F (2011). The deletion of exon 3 in the cardiac ryanodine receptor is rescued by beta strand switching. Structure.

[23] Gong D (2019). Modulation of cardiac ryanodine receptor 2 by calmodulin. Nature.

[24] Peng, W. et al. Structural basis for the gating mechanism of the type 2 ryanodine receptor RyR2. *Science* **354**, aah5324 (2016). 10.1126/science.aah5324 27708056

[25] Willegems K, Efremov RG (2018). Influence of lipid mimetics on gating of ryanodine receptor. Structure.

[26] Bai XC, Yan Z, Wu J, Li Z, Yan N (2016). The central domain of RyR1 is the transducer for long-range allosteric gating of channel opening. Cell Res..

[27] Gillard EF (1991). A substitution of cysteine for arginine 614 in the ryanodine receptor is potentially causative of human malignant hyperthermia. Genomics.

[28] Fujii J (1991). Identification of a mutation in porcine ryanodine receptor associated with malignant hyperthermia. Science.

[29] Jiang D (2008). Reduced threshold for luminal Ca^2+^ activation of RyR1 underlies a causal mechanism of porcine malignant hyperthermia. J. Biol. Chem..

[30] Balshaw DM, Xu L, Yamaguchi N, Pasek DA, Meissner G (2001). Calmodulin binding and inhibition of cardiac muscle calcium release channel (ryanodine receptor). J. Biol. Chem..

[31] Ikemoto T, Iino M, Endo M (1995). Enhancing effect of calmodulin on Ca(2+)-induced Ca^2+^ release in the sarcoplasmic reticulum of rabbit skeletal muscle fibres. J. Physiol..

[32] Tripathy A, Xu L, Mann G, Meissner G (1995). Calmodulin activation and inhibition of skeletal muscle Ca^2+^ release channel (ryanodine receptor). Biophys. J..

[33] Buratti R, Prestipino G, Menegazzi P, Treves S, Zorzato F (1995). Calcium dependent activation of skeletal muscle Ca^2+^ release channel (ryanodine receptor) by calmodulin. Biochem Biophys. Res. Commun..

[34] Fuentes O, Valdivia C, Vaughan D, Coronado R, Valdivia HH (1994). Calcium-dependent block of ryanodine receptor channel of swine skeletal muscle by direct binding of calmodulin. Cell Calcium.

[35] Oo YW (2015). Essential role of calmodulin in RyR inhibition by dantrolene. Mol. Pharmacol..

[36] Diszhazi G (2019). Dantrolene requires Mg(2+) and ATP to inhibit the ryanodine receptor. Mol. Pharm..

[37] Moore CP (1999). Apocalmodulin and Ca^2+^ calmodulin bind to the same region on the skeletal muscle Ca^2+^ release channel. Biochemistry.

[38] Lau K, Chan MM, Van Petegem F (2014). Lobe-specific calmodulin binding to different ryanodine receptor isoforms. Biochemistry.

[39] Zhang H, Zhang JZ, Danila CI, Hamilton SL (2003). A noncontiguous, intersubunit binding site for calmodulin on the skeletal muscle Ca^2+^ release channel. J. Biol. Chem..

[40] McCarthy MR, Savich Y, Cornea RL, Thomas DD (2020). Resolved structural states of calmodulin in regulation of skeletal muscle calcium release. Biophys. J..

[41] Iyer, K. A. et al. Structural mechanism of two gain-of-function cardiac and skeletal RyR mutations at an equivalent site by cryo-EM. *Sci. Adv.* **6**, eabb2964 (2020). 10.1126/sciadv.abb2964 PMC7439390 32832689

[42] Gabriel, S., Ziaugra, L. & Tabbaa, D. SNP genotyping using the Sequenom MassARRAY iPLEX platform. *Curr. Protoc. Hum. Genet.* **Chapter 2**, 12 (2009). 10.1002/0471142905.hg0212s60 19170031

[43] Lee HB, Xu L, Meissner G (1994). Reconstitution of the skeletal muscle ryanodine receptor-Ca^2+^ release channel protein complex into proteoliposomes. J. Biol. Chem..

[44] Schoenmakers TJ, Visser GJ, Flik G, Theuvenet AP (1992). CHELATOR: an improved method for computing metal ion concentrations in physiological solutions. Biotechniques.

[45] Shomer NH, Mickelson JR, Louis CF (1994). Ion selectivity of porcine skeletal muscle Ca^2+^ release channels is unaffected by the Arg615 to Cys615 mutation. Biophys. J..

[46] Punjani A, Rubinstein JL, Fleet DJ, Brubaker MA (2017). cryoSPARC: algorithms for rapid unsupervised cryo-EM structure determination. Nat. Methods.

[47] Zivanov, J. et al. New tools for automated high-resolution cryo-EM structure determination in RELION-3. *Elife* **7**, e42166 (2018). 10.7554/eLife.42166 PMC6250425 30412051

[48] Quick AP (2017). SPEG (striated muscle preferentially expressed protein kinase) is essential for cardiac function by regulating junctional membrane complex activity. Circ. Res..

[49] Terentyev D, Hamilton S (2016). Regulation of sarcoplasmic reticulum Ca(2+) release by serine-threonine phosphatases in the heart. J. Mol. Cell Cardiol..

[50] Respress JL (2012). Role of RyR2 phosphorylation at S2814 during heart failure progression. Circ. Res..

[51] Afonine PV (2018). Real-space refinement in PHENIX for cryo-EM and crystallography. Acta Crystallogr D. Struct. Biol..

[52] Terwilliger TC, Ludtke SJ, Read RJ, Adams PD, Afonine PV (2020). Improvement of cryo-EM maps by density modification. Nat. Methods.

[53] Pettersen EF (2004). UCSF Chimera—a visualization system for exploratory research and analysis. J. Comput. Chem..

[54] Ather S (2013). Inhibition of CaMKII phosphorylation of RyR2 prevents inducible ventricular arrhythmias in mice with Duchenne muscular dystrophy. Heart Rhythm.

[55] Li N (2012). Inhibition of CaMKII phosphorylation of RyR2 prevents induction of atrial fibrillation in FKBP12.6 knockout mice. Circ. Res..

