# Supplementary material for: Pathological conformations of disease mutant Ryanodine Receptors revealed by cryo-EM
Source: Nat Commun. 2021 Feb 5;12:807. doi: 10.1038/s41467-021-21141-3 (PMC7864917; doi:10.1038/s41467-021-21141-3)
Supplement: Supplementary file 1 — Supplementary Information [file 41467_2021_21141_MOESM1_ESM.pdf]

Woll et al. - Supplementary Figures, Tables, and References

Supplementary Figure 1

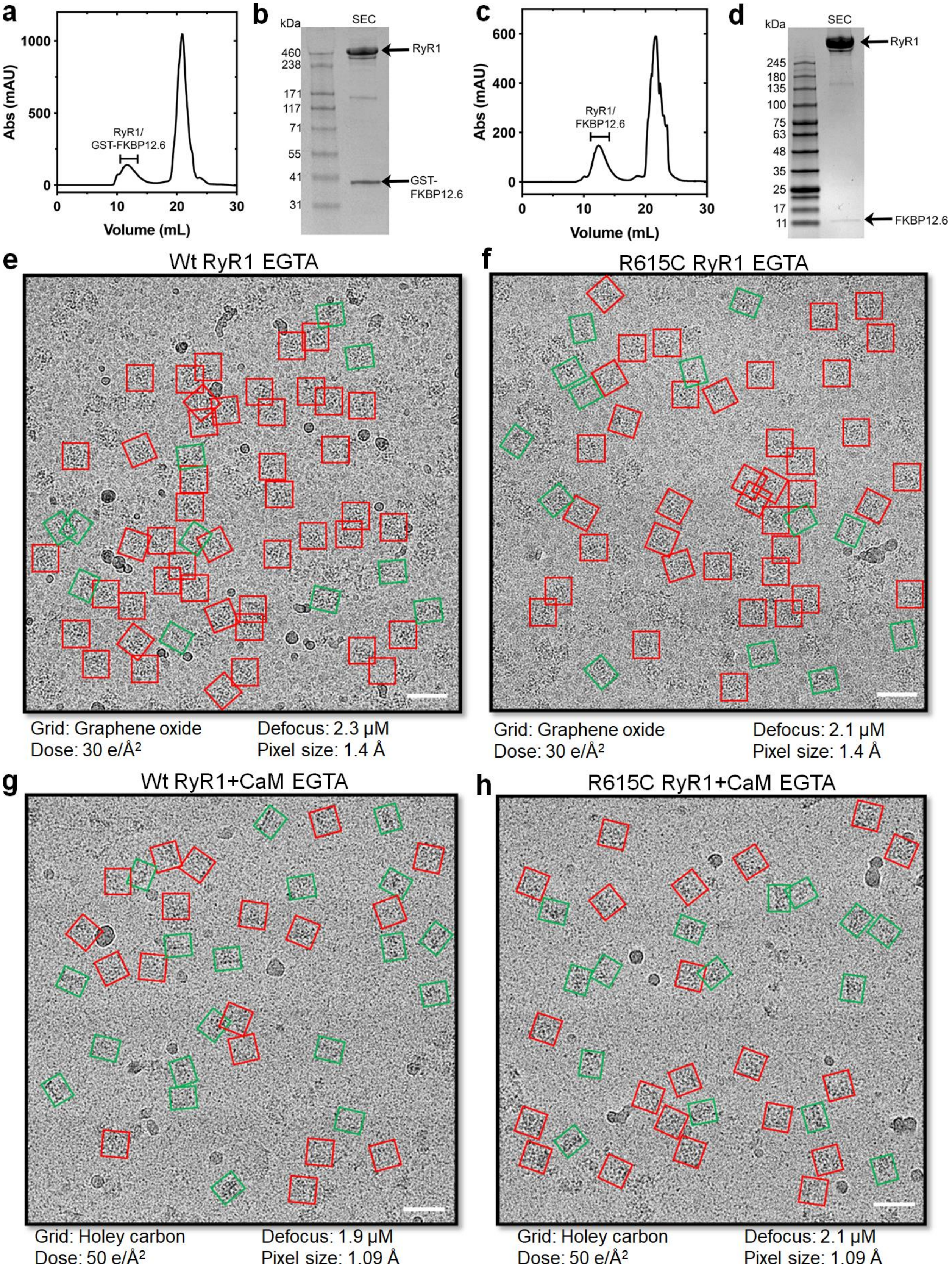

Supplementary Figure 1. Quality of the purified pig RyR1 (pRyR1). Purification method of the GST-FKBP12.6/RyR1 complex maintaining the GST linkage including **a,** Superose 6 size exclusion chromatogram and **b,** representative corresponding Coomassie blue stained SDS-PAGE of size exclusion chromatography (SEC) peak eluting at ~12mL. Repeats with independent batches show a similar result. Purification method of the FKBP12.6/RyR1 complex employing TEV cleavage to remove the GST tag, including **c,** Superose 6 size exclusion chromatogram and **d,** corresponding representative Coomassie blue stained SDS-PAGE of SEC peak eluting at ~13mL. Repeats with independent batches show a similar result. **e-h,** Comparison between representative motion corrected micrographs of WT pRyR1 in EGTA (total number of movies = 1986), WT pRyR1 in EGTA and calmodulin (Wt RyR1+apoCaM, total number of movies = 1491)), R615C in EGTA (total number of movies = 2512), and R615C and calmodulin in EGTA (total number of movies = 1949) on holey carbon grids with or without graphene oxide (GO) film at different electron dose and pixel size. For clarity we have highlighted a few of the RyR1 particles with red boxes representing top/bottom views and green boxes representing side views. The white scale bars correspond to 400Å.

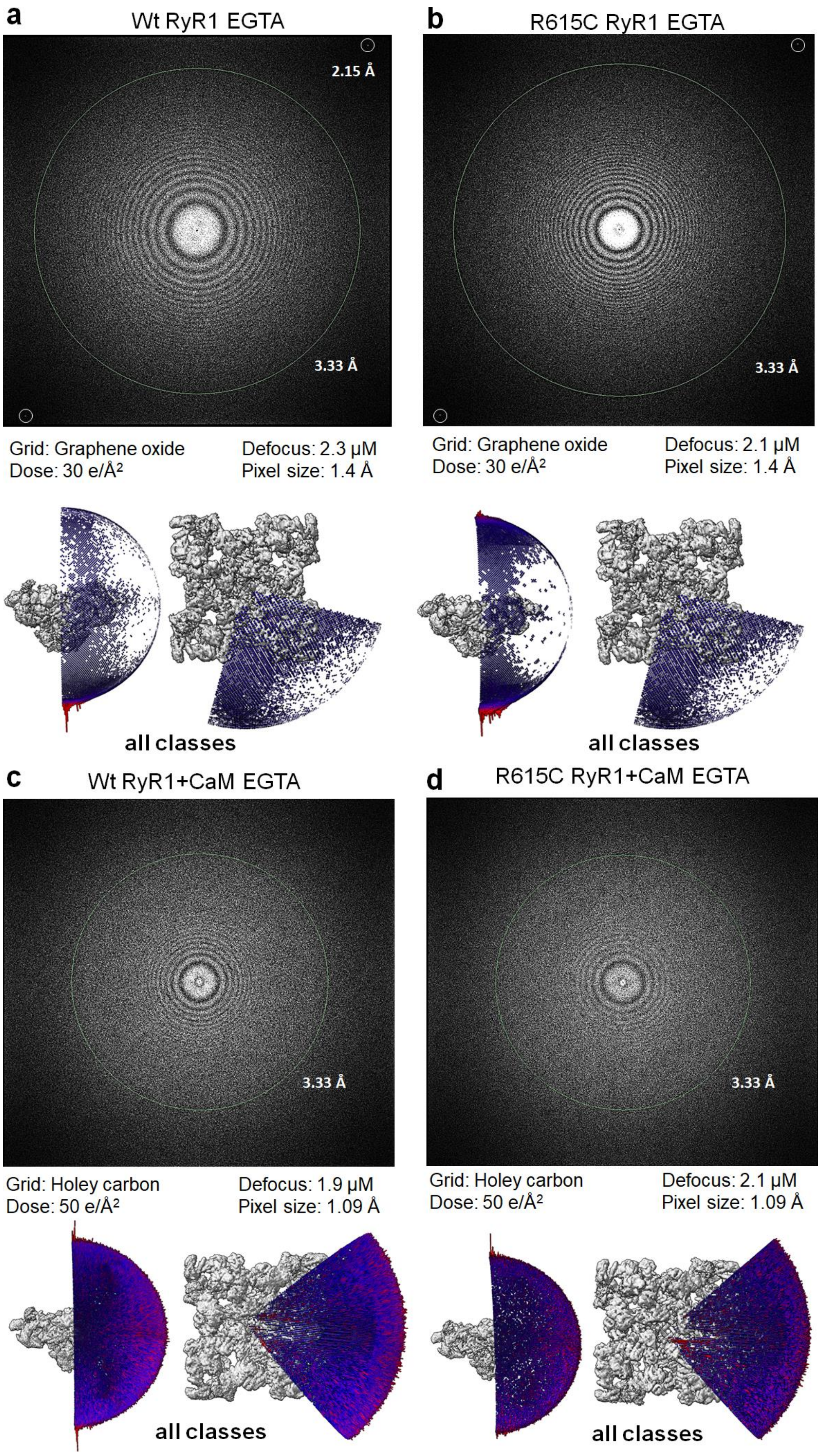
a
Wt RyR1 EGTA
2.15 Å
3.33 Å
Grid: Graphene oxide
Dose: 30 e/Å²
Defocus: 2.3 μM
Pixel size: 1.4 Å
all classes
b
R615C RyR1 EGTA
3.33 Å
Grid: Graphene oxide
Dose: 30 e/Å²
Defocus: 2.1 μM
Pixel size: 1.4 Å
all classes
c
Wt RyR1+CaM EGTA
3.33 Å
Grid: Holey carbon
Dose: 50 e/Å²
Defocus: 1.9 μM
Pixel size: 1.09 Å
all classes
d
R615C RyR1+CaM EGTA
3.33 Å
Grid: Holey carbon
Dose: 50 e/Å²
Defocus: 2.1 μM
Pixel size: 1.09 Å
all classes

Supplementary Figure 2. Power spectra and orientation distribution. Background subtracted power spectra of data sets collected with graphene oxide grids for **a.** WT RyR1 in EGTA and **b.** R615C RyR1 in EGTA and for datasets collected with holey carbon grids for **c.** WT RyR1 with calmodulin in EGTA and **d.** R615C RyR1 with calmodulin in EGTA. Discrete diffraction spots for graphene oxide can be observed beyond Nyquist, as reported before[1], which appear at between 2.1-2.2 Å (depending on tilt, aberration and defocus range). The Euler angle distributions of all particles used for calculating the 3D reconstructions with imposed C4 symmetry for each collected dataset are shown below for each dataset. The height and color of bars represent the relative angle distribution of reference for orientation with the smallest number in dark blue and greatest number in red.

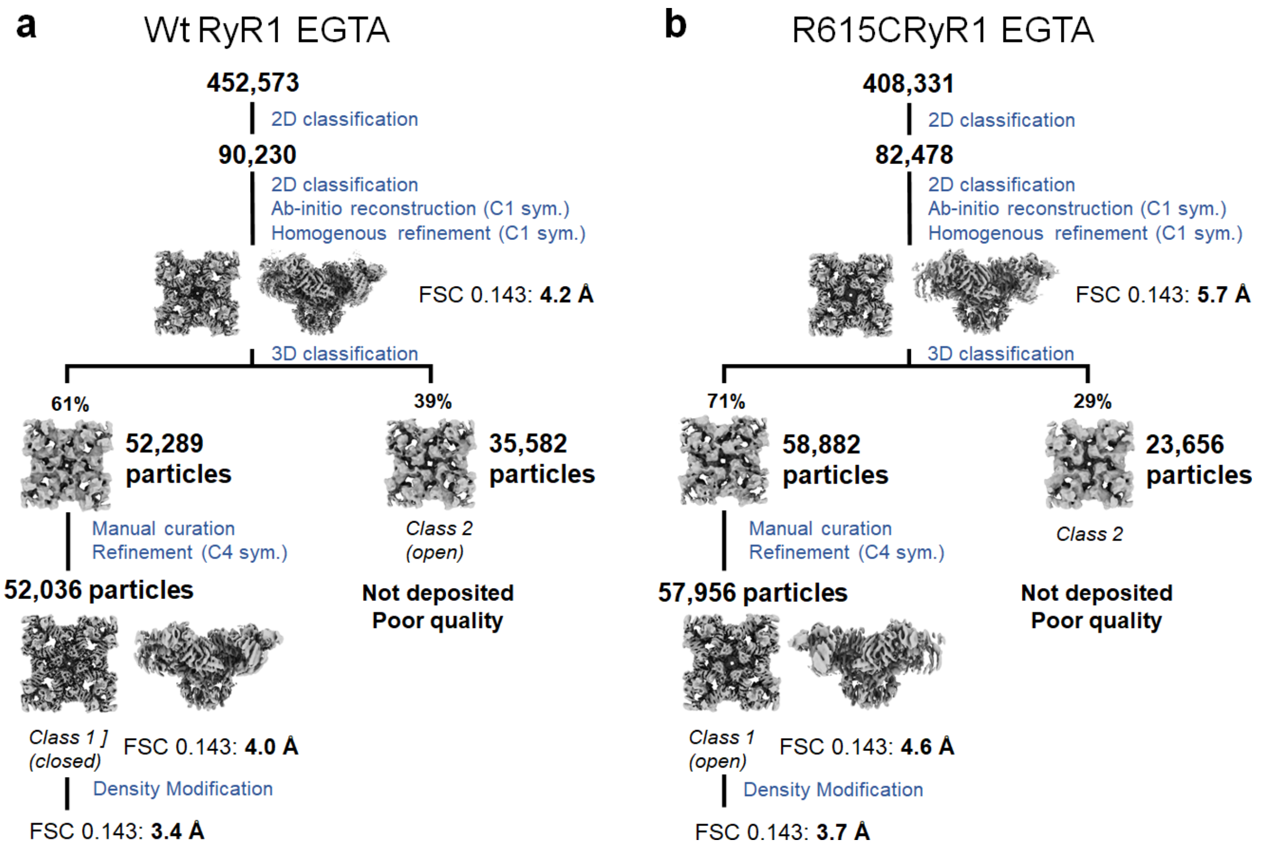

Supplementary Figure 3. Global refinement procedures and 3D classification for structures without apoCaM a. WTpRyR1 **b.** R615C RyR1. Both are in the presence of 5mM EGTA and were processed with CryoSPARC v2.9/2.12.4. All resolutions reported in this figure are using the FSC=0.143 criterion determined by CryoSPARC (original map) or using Phenix *Resolve* density modification. For both datasets, the 2nd class suffered from poor quality particles that did not result in high-quality maps. Note that the resolutions after Density Modification cannot be directly compared to the one before as shown here, because different masks were applied. In order to judge the relative improvement due to density modification, please see Supplementary Fig. 4c,d.

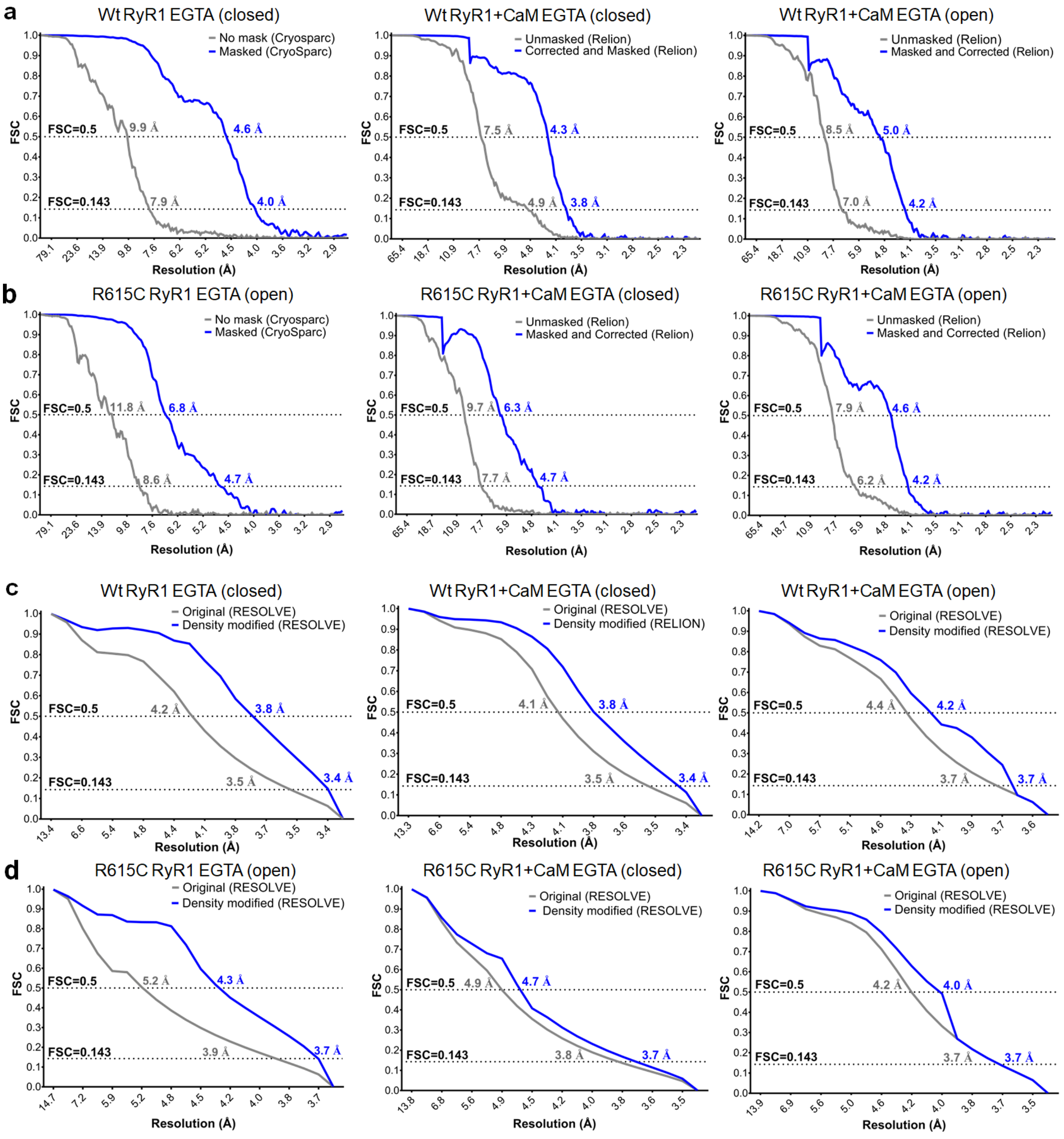

Supplementary Figure 4. Fourier Shell Correlation curves. Masked and unmasked Fourier Shell Correlation (FSC) curves for **a.** wildtype (Wt RYR1) and **b.** R615C mutant (R615C RyR1) RyR1 EGTA conditions in the absence and presence of apo-calmodulin (+CaM) as reported by cryoSPARC and RELION. **c.** FSC curves reported from Phenix RESOLVE for the original and density modified maps for each dataset. Note that these maps are rescaled in Phenix RESOLVE. Intercepts are shown at 0.143 and 0.5 FSC cut-offs.

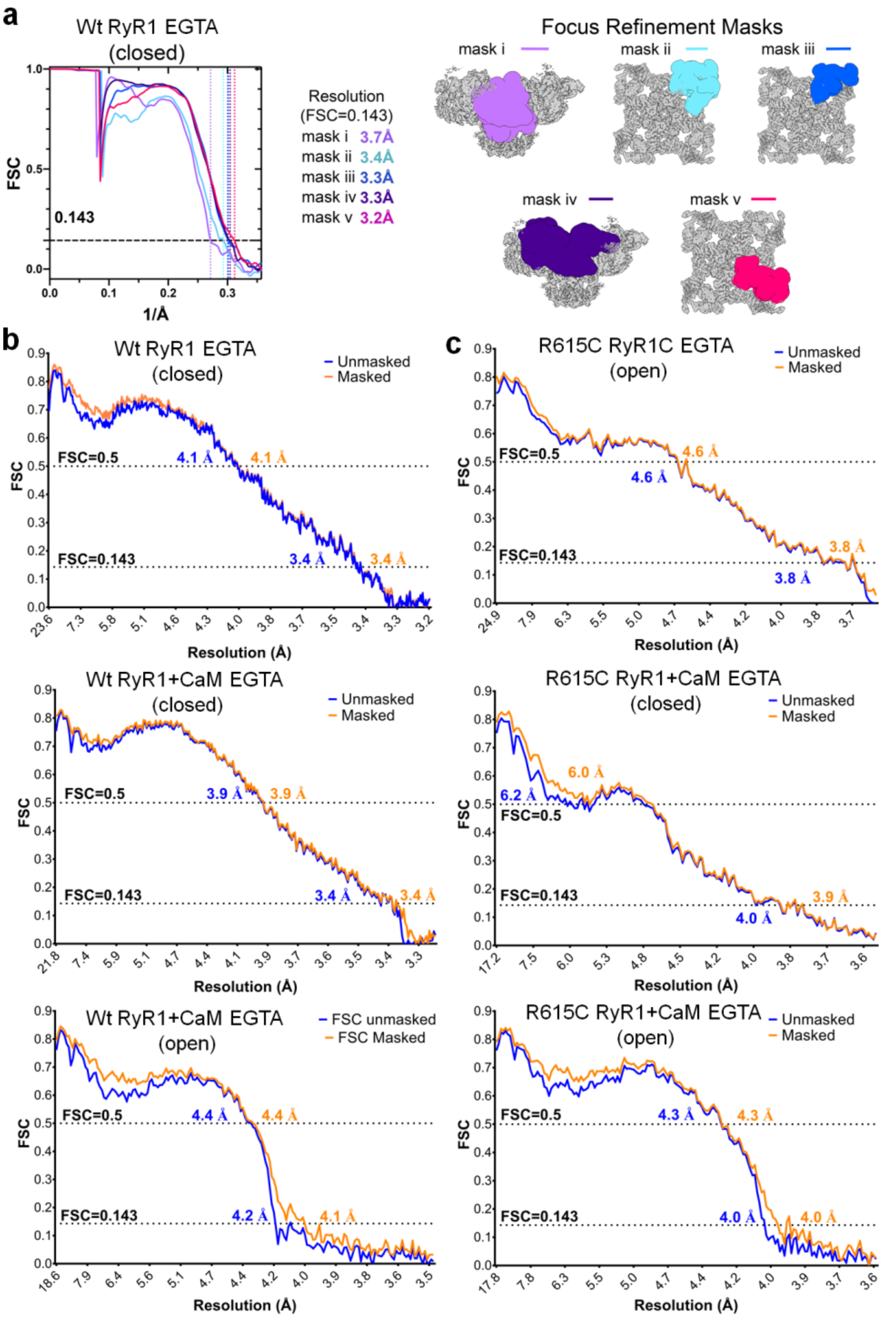

Supplementary Figure 5. Focused refinement and map to model correlation. a. *Left*, Representative FSC curves of Wildtype RyR1 EGTA condition focused refinement using RELION 3.0-beta without particle subtraction and 'gold standard' protocol (FSC = 0.143, black dash) for mask i (mauve), mask ii (cyan), mask iii (blue), mask v (pink), mask iv (purple). *Right*, Representative masks applied during focused refinement. **b, c.** Map to model FSC curve for all 6 structures presented in this manuscript. Curves were calculated using Phenix. Data point were pruned in GraphPad Prism to average out the data point for every 100 rows (k=100).

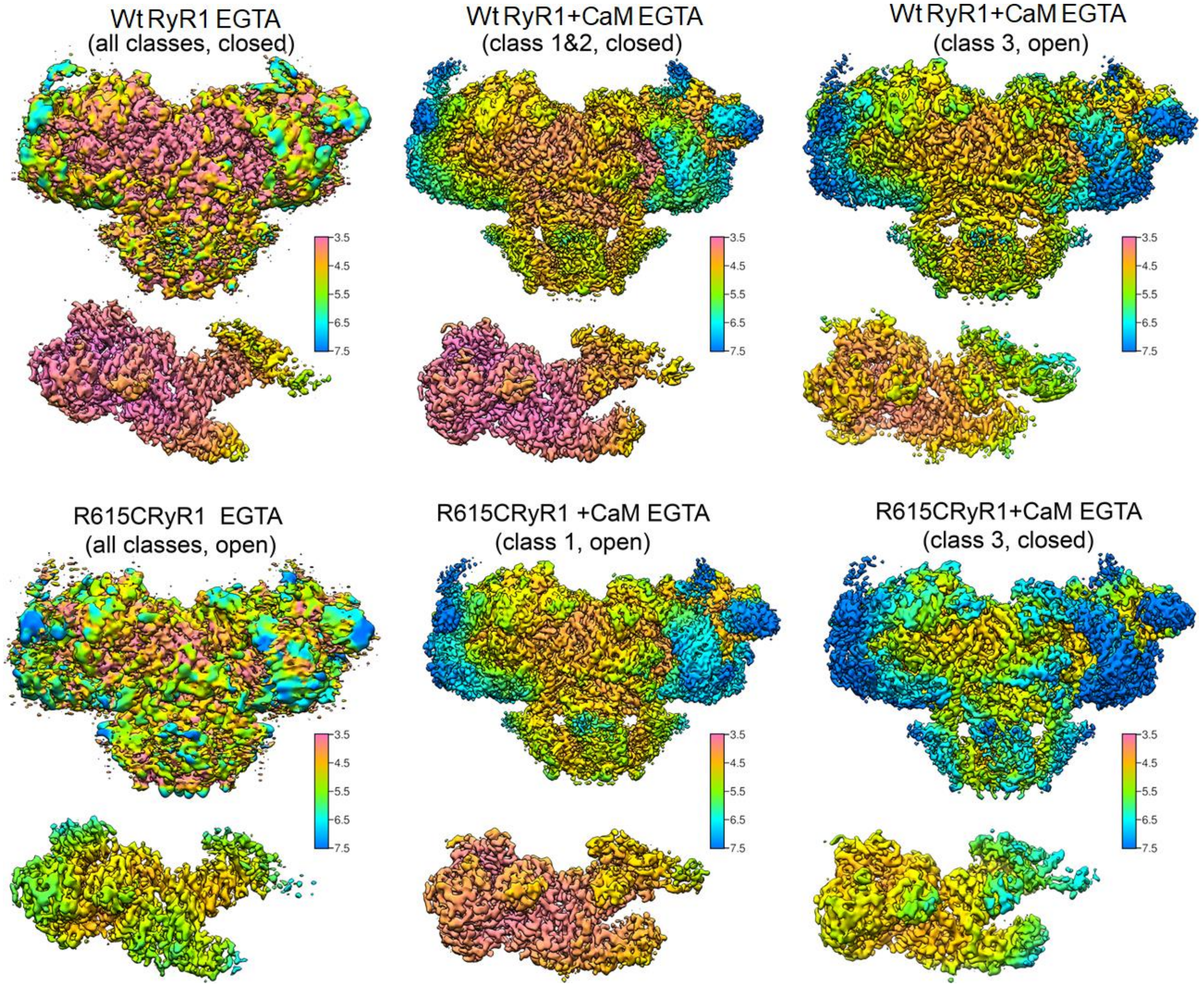

Supplementary Figure 6. Local resolutions. Class reconstructions and focused refinement with mask iv colored by local resolution from 3.5Å (pink) to 7.5Å (blue) using RELION3.0-beta for wildtype (Wt RyR1) and R615C mutant (R615CRyR1) RyR1 EGTA conditions with (+CaM) and without calmodulin, viewed from the plane of the SR membrane (σ=3-5).

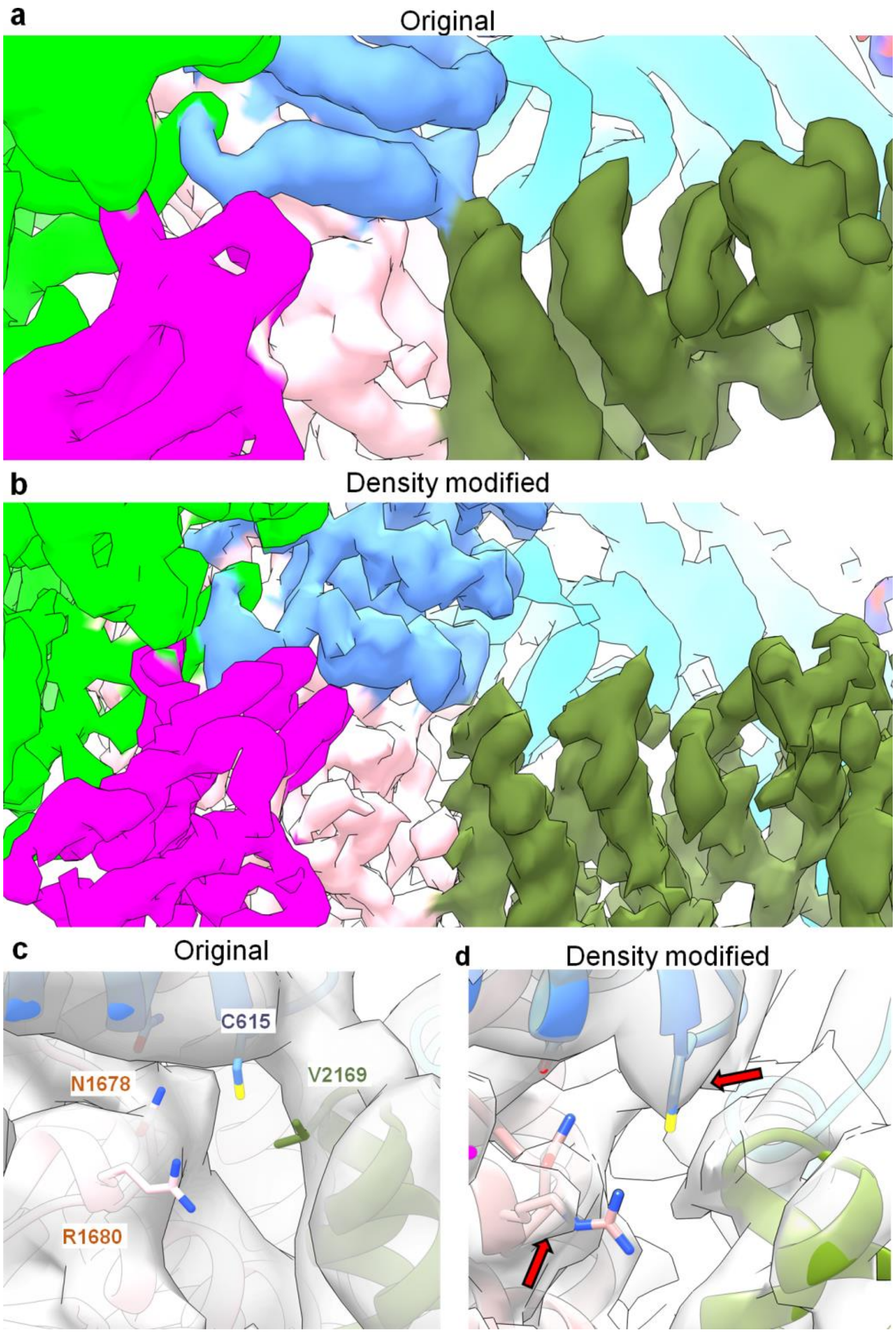

Supplementary Figure 7. Improvements through density modification. a. Original cryoSPARC-derived cryo-EM map for R615C pRyR1 (open, no CaM) highlighting areas around the mutation site, and **b.** the same region shown for the density modified map calculated by PHENIX RESOLVE. Colour scheme is identical to Fig. 2a. **c.** Zoomed in view of the R615C mutation site for original and **d.** density modified map, showing a detail around residue 615. Arrows indicate density improvements not observed in the original map. Sigma (σ) contour level set to ~4.

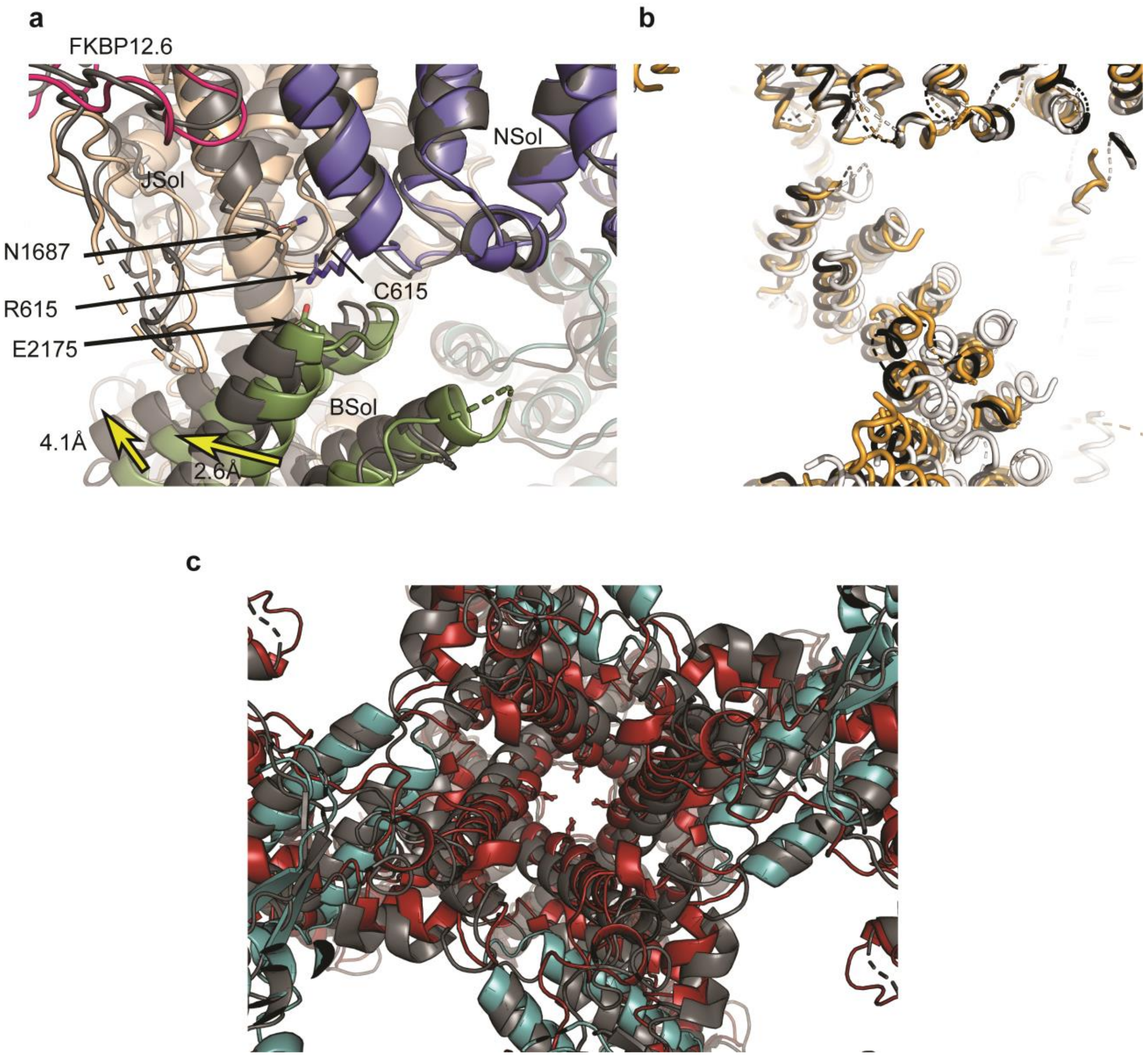

Supplementary Figure 8. Effects of the R615C mutation on the conformation. a. Superposition, based on the Nsol region, of WT (colors) and R615C pRyR1 (dark gray), both in the absence of apoCaM. Arg 615 sits at a junction between the Nsol, Jsol, and Csol regions. Nearby residues are labeled. The R615C mutation results in movements of the JSol and the Bsol, relative the Nsol region. **b.** Superposition, based on the Nsol region, of WT pRyR1 (white), R615C pRyR1 (black), and activated rabbit RyR1 (orange) in complex with ATP, Ca^{2+} and caffeine (PDB ID 5TAL). The position of the R615C Bsol region, relative to the Nsol, is closer to the activated rabbit RyR1 (orange), but is not the same: it is hyper-extended compared to the activated rabbit RyR1. **c.** Superposition of the pore-forming region of WT (colors) and R615C pRyR1 (black), with Ile4935 shown in sticks. The pore of R615C pRyR1 is in an open conformation.

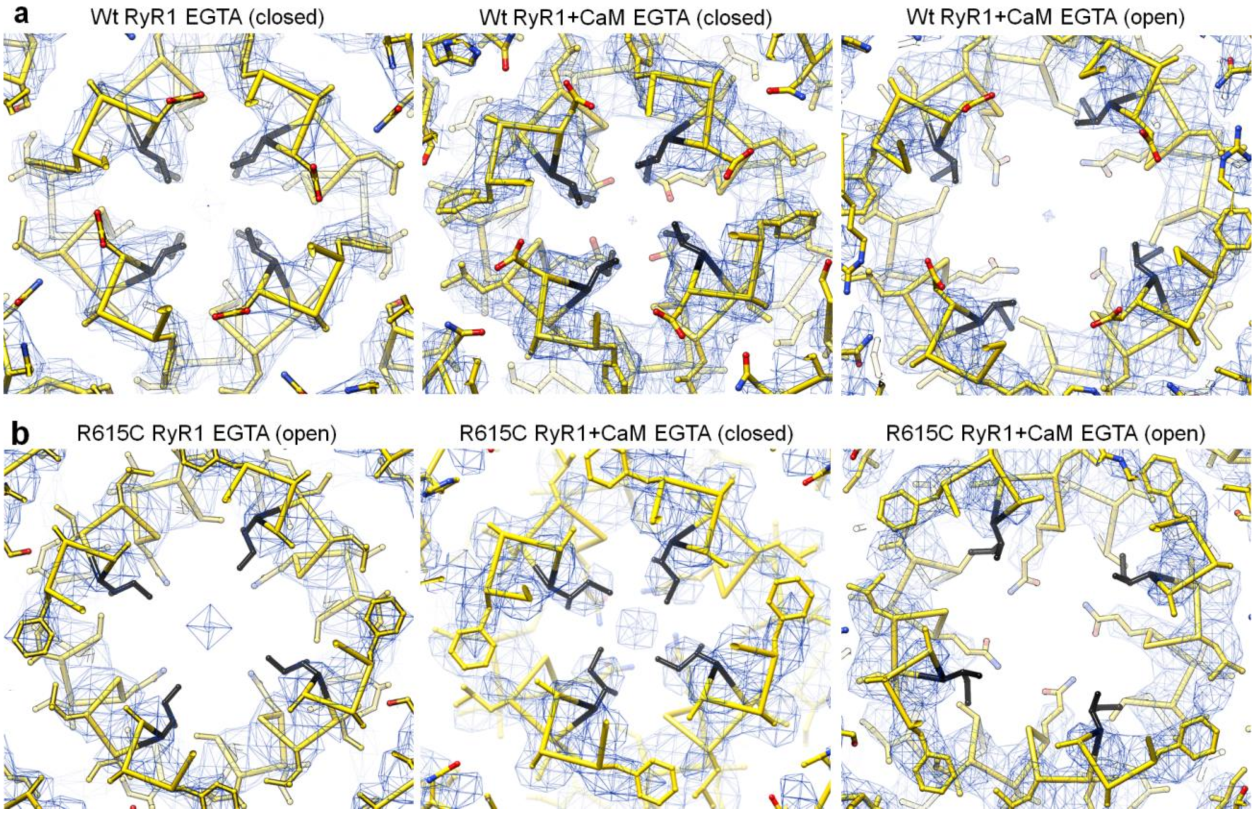

Supplementary Figure 9. Cryo-EM density maps in the pore regions. Cryo-EM maps (density modified or composite maps calculated from focused refinement) highlighting the region around the Ile4935 residue (black) and nearby RyR1 residues (yellow) for **a.** WT and **b**. R615C structures, respectively. Sigma (σ) contour level set to ~3.6 for all maps.

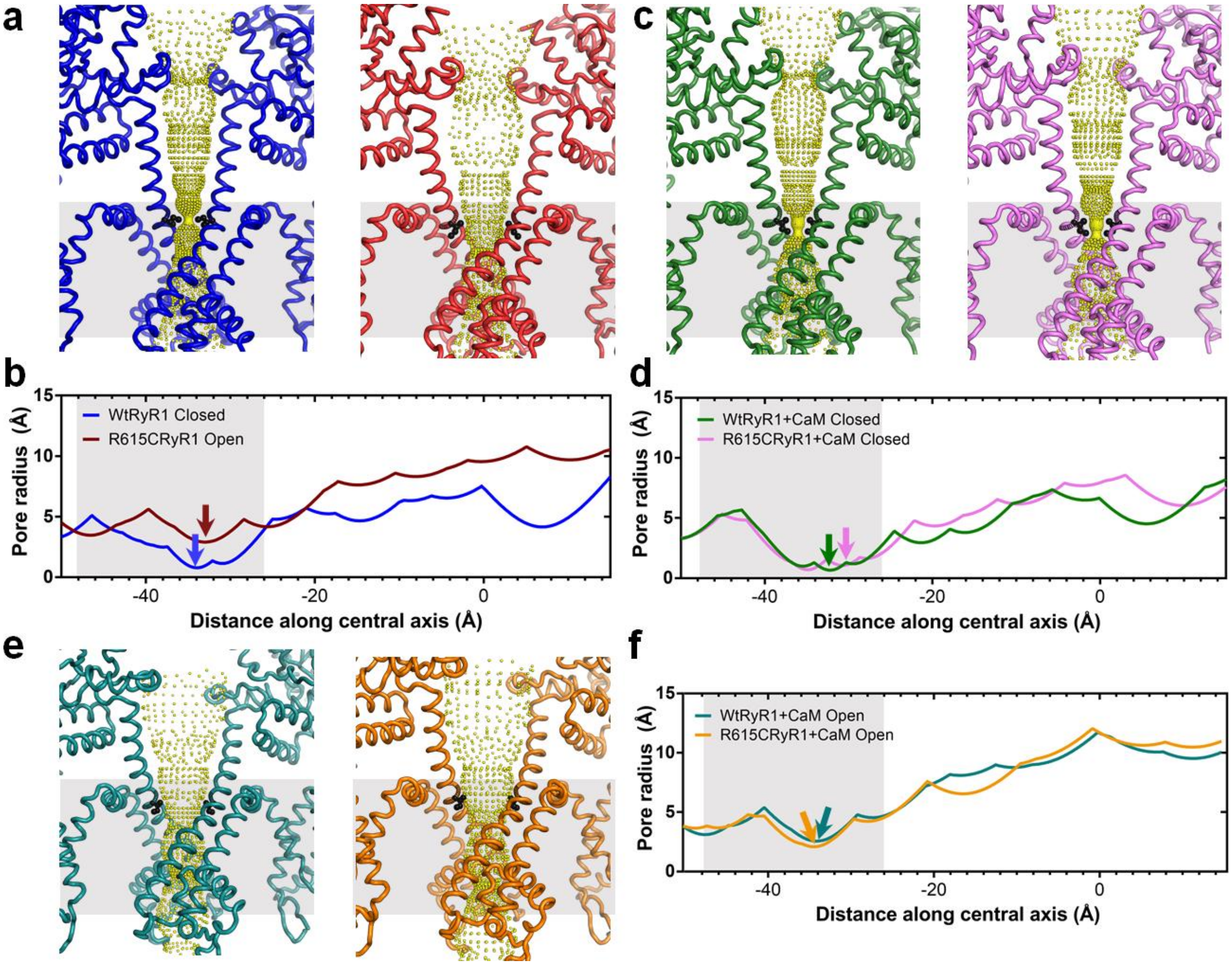

Supplementary Figure 10. Pore radii. a. Models of transmembrane domain helices S5-6 and CTD with HOLE pore diameter estimations (*yellow dots*) for WT pRyR1 (blue, closed) and R615C pRyR1 (open, red). Only 2 subunits are shown for clarity. The hydrophobic gating residue Ile4935 is indicated by black spheres. **b.** Corresponding graph showing the pore radius. The position of Ile4935 is indicated by arrows. **c,d.** Similar plots for closed WT RyR1+apoCaM (green) and closed R615C pRyR1 + apoCaM (violet). **e,f.** Similar plots for open WT pRyR1+apoCaM (teal) and open R615C pRyR1+apoCaM (orange). In every plot, the transmembrane region is highlighted by gray shading. Although the side chain density for Ile4935 is poor in the lowest-resolution structures, the state (open vs closed) of the pore can also be reliably obtained from the position of the S6 helices, which also constrain the position of the side chain. Although the position may not be as accurate compared to the higher-resolution structures, we have included the side chain in each structure in order to generate the hole plots.

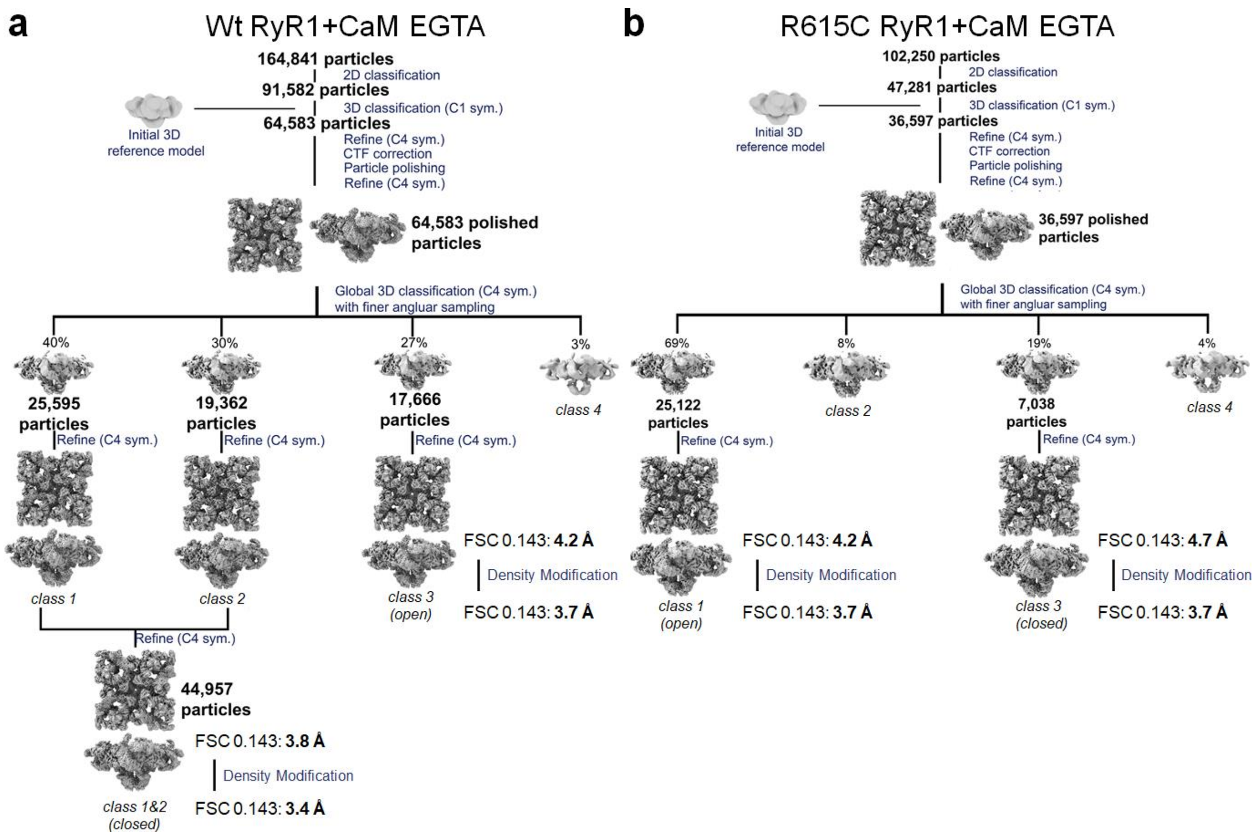

Supplementary Figure 11. Global refinement procedures and 3D classification for structures with apoCaM. a. Global classification and refinement procedures used for Wt pRyR1 in the presence of 5mM EGTA and calmodulin (Wt RyR1+CaM EGTA). **b.** Global classification and refinement procedures used for R615C pRyR1 in the presence of 5mM EGTA and calmodulin (R615CRyR1+CaM EGTA). All resolutions reported are using the FSC=0.143 criterion using RELION and Phenix *Resolve* post density modification. However, the resolutions before and after density modification here cannot be directly compared, as different masks were applied. For a better estimate of the relative improvement due to density modification, please see Fig. 4c,d.

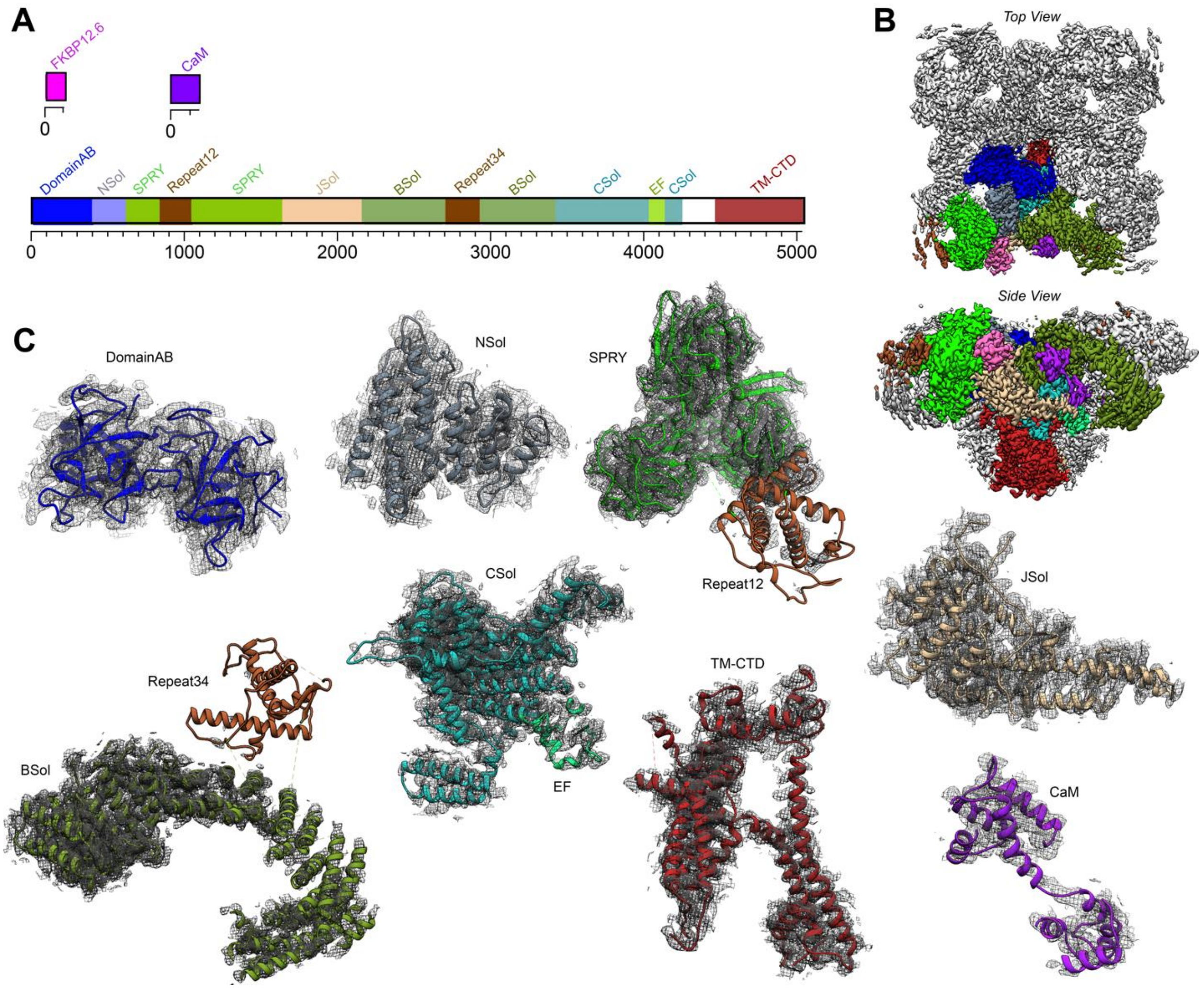

Supplementary Figure 12. Representative cryo-EM density maps. a. Protein and domain map for FKBP12.6, CaM, and pRyR1 complexes (CaM, Calmodulin; NSol, N-solenoid; BSol, B-solenoid; CSol, C-solenoid; EF, EF-hand; JSol, J-solenoid; TM-CTD, Transmembrane and Carboxy terminal domain. **b**. Top and side views of wild type pRyR1 closed state with calmodulin with one subunit colored according to the scheme in panel A. **c.** Representations of cryo-EM map and model for the wild type pRyR1 + apoCaM in the closed state for various regions outlined in panel a, contoured at σ=3-4.

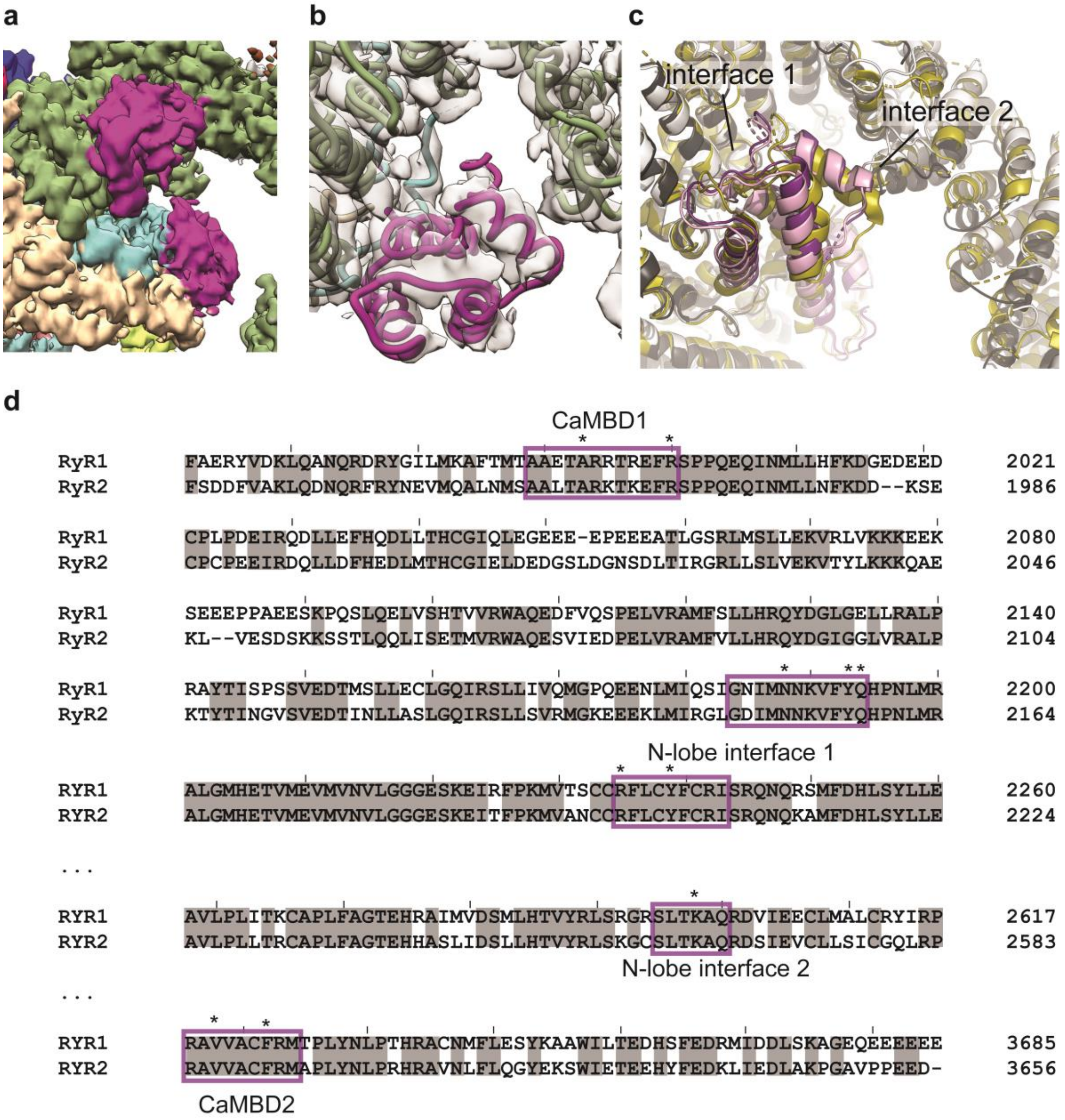

Supplementary Figure 13. ApoCaM binding regions. a. Map of wild type closed pRyR1+apoCaM, showing the area around apoCaM. Color coding as shown in Supplementary Fig. 12. **b.** Map and model around the apoCaM N-lobe. **c.** Superposition, based on interface 1, of closed WT pRyR1+apoCaM (white, light pink), open WT pRyR1+apoCaM (black, dark purple), and pRyR2 + apoCaM (PDB 6JI8, gold). This shows that apoCaM does not cause the degree of bending of the BsoI of pRyR2 as seen for open WT pRyR1+apoCaM **d.** Sequence alignment of distinct regions in pRyR1 and pRyR2. Gray shades indicate conserved residues. Purple boxes indicate regions that bind apoCaM. The most prominent residues involved in binding are indicated by asterisks. The high sequence conservation of the interacting residues suggests that the structural differences, observed for pRyR1 and pRyR2 may be due to general sequence differences scattered across the BsoI region, which influence its ability to bend upon binding of apoCaM.

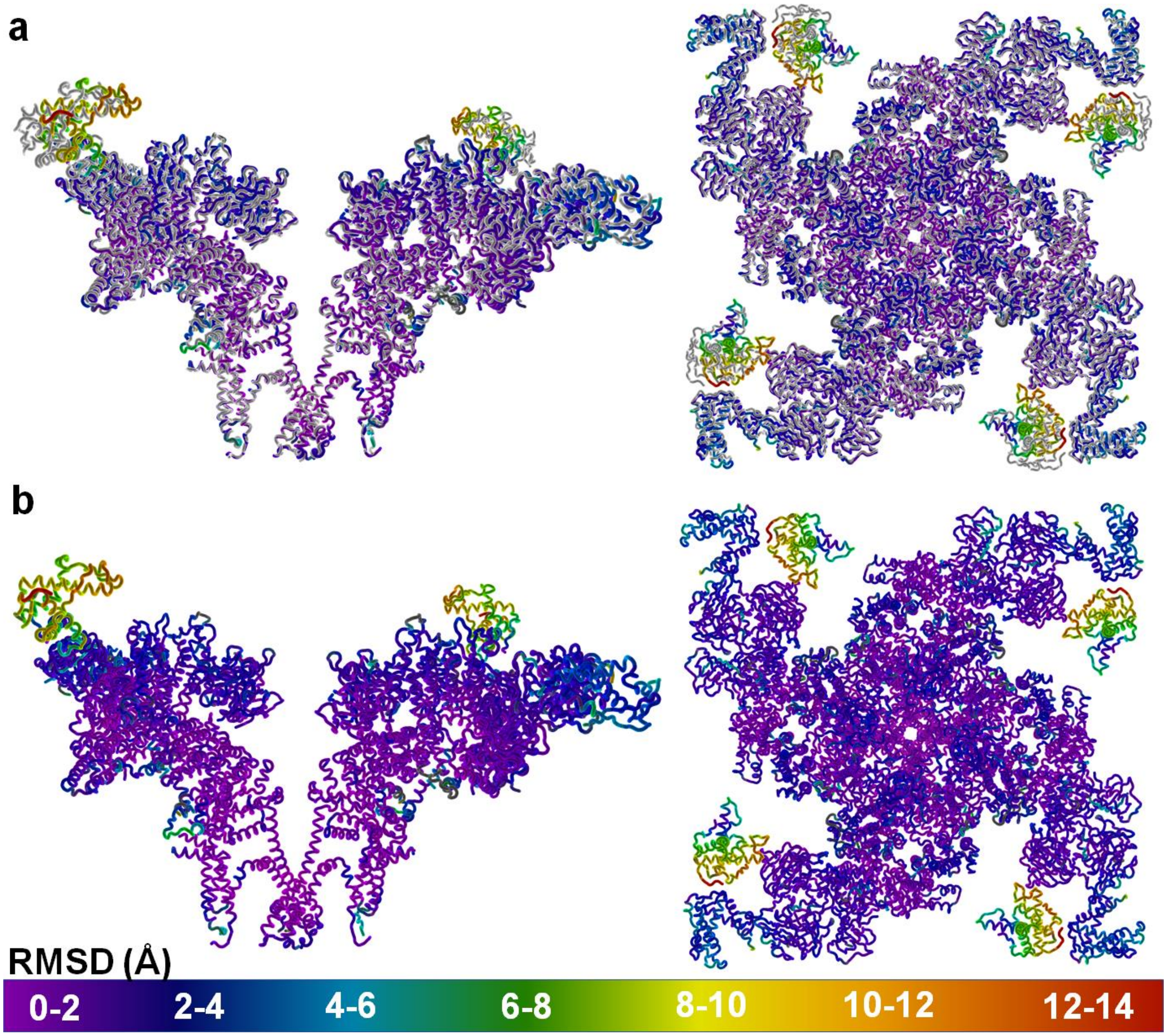

Supplementary Figure 14. Comparison between rabbit and pig RyR1. **a**. Ribbon representation of sequence independent structural superposition between closed WT pig RyR1 EGTA condition (white) and previously published WT rabbit RyR1 structure (coloured) determined in a similar condition (PDB: 5TB0). The colouring scheme for root mean square deviations (RMSD) are shown below the figure in the colour bar. Regions with unknown sequence assignments were not considered.

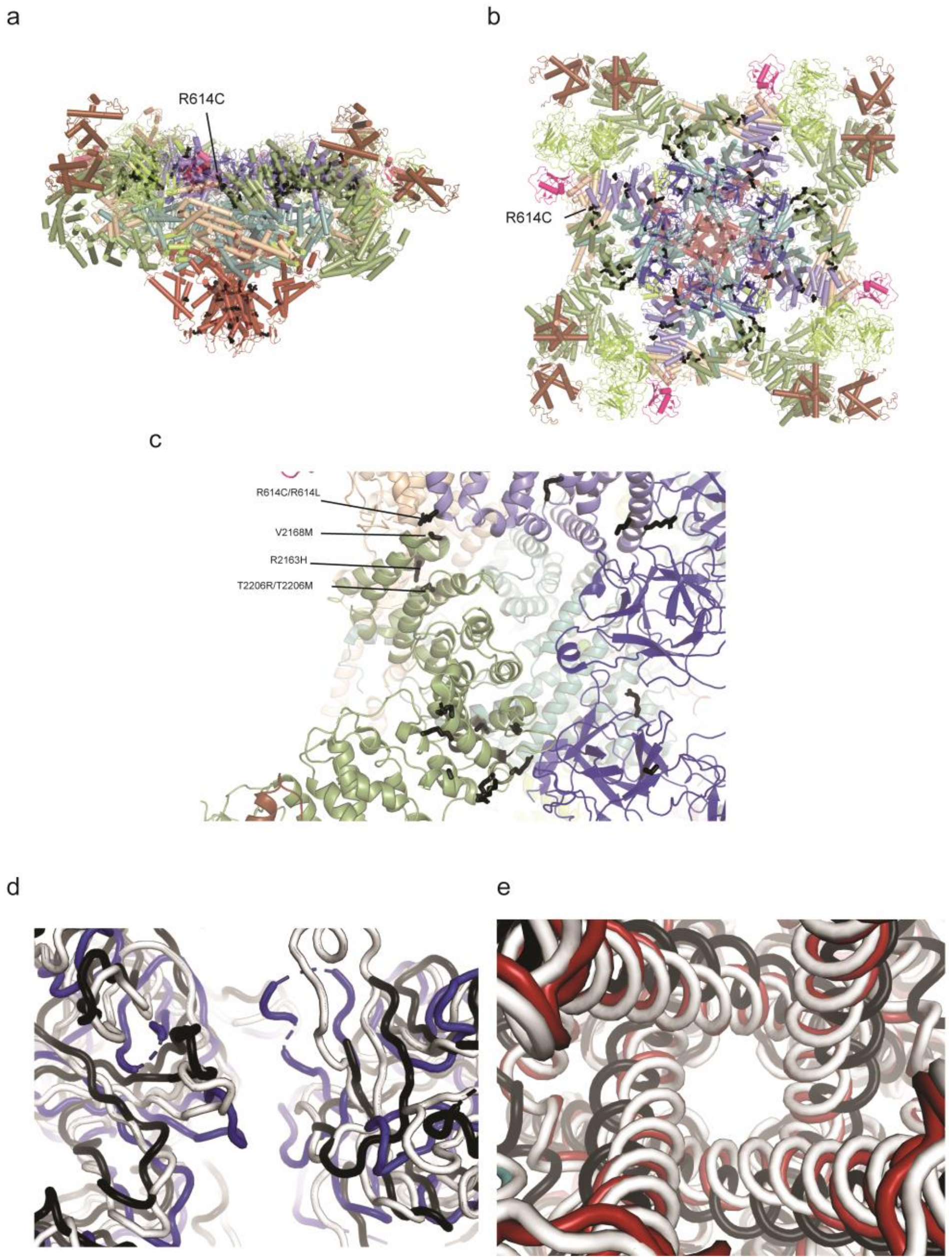

Supplementary Figure 15. Disease mutations in RyR1. Locations of MH mutations considered diagnostic (www.emhg.org). **a.** Side view of RyR1 within the plane of the SR membrane. **b.** Top view facing the SR. **c.** Detail showing mutations close to the human R614C mutation (equivalent to the porcine R615C in this study). Domains are colored as in Figure 1. The positions of residues targeted by mutations are indicated by black sticks. The location of R614C is indicated, as well as selected mutations in its vicinity in panel c. **d.** Comparison of the NTD area, showing the inter-subunit interface, for structures of WT pRyR1 (colors), R615C pRyR1 (black), and R164C rabbit RyR1 (PDB 6WOT, white). This shows that the effect of the R164C mutation in RyR1 is more subtle, with a conformation intermediate between WT and R615C. **e.** Same superposition, showing the pore region, indicating the only the R615C (black) results in predominantly open channels.

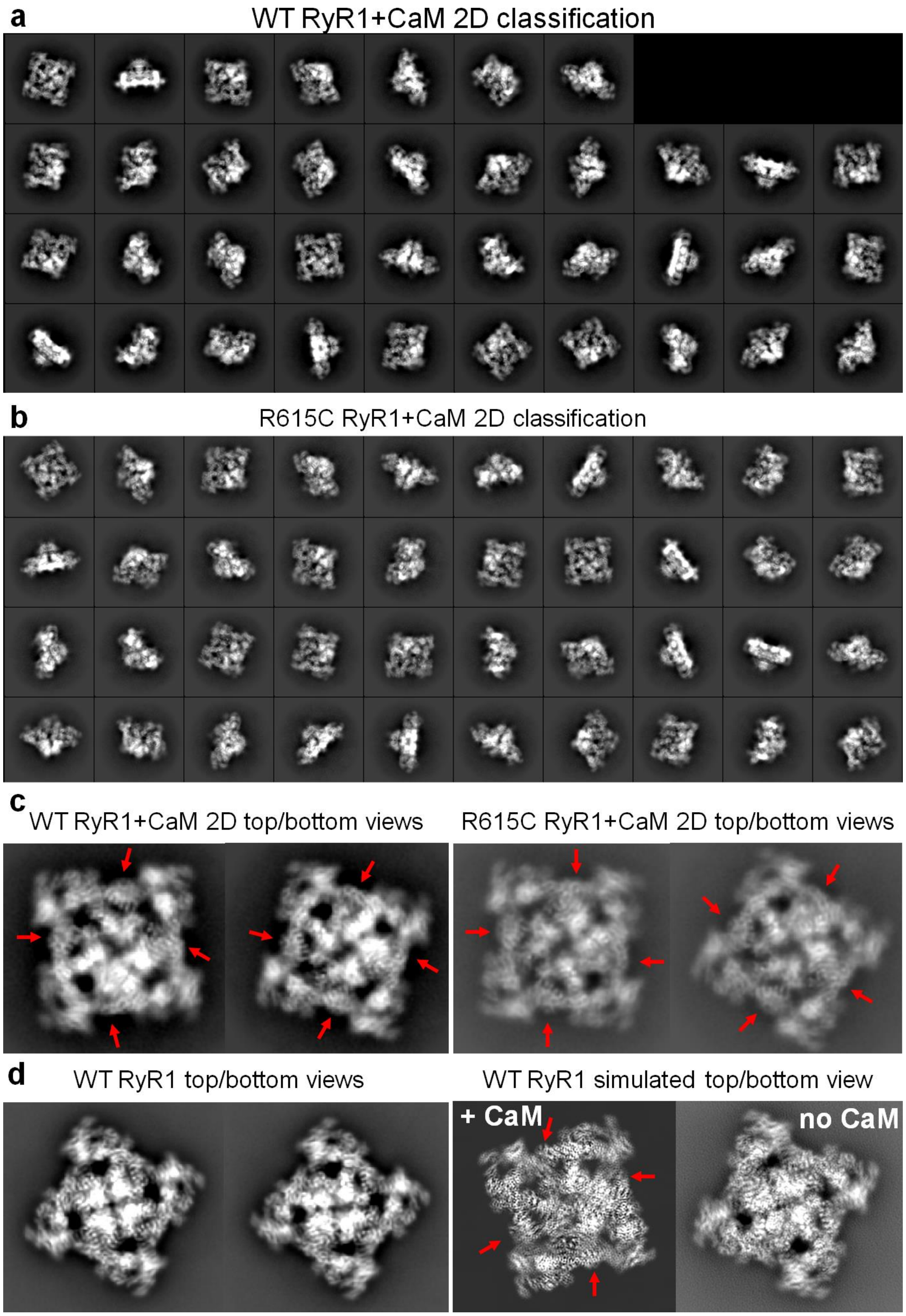

Supplementary Figure 16. 2D classification results. **a.** WT RyR1+apoCaM and **b.** R615C RyR1+apoCaM. **c.** Top and bottom views obtained from 2D classification for WT and for R615C datasets in presence of apoCaM. Red arrows indicate bulges for apoCaM. **d.** Two bottom views of WT RyR1 (no CaM) compared to the simulated projections of RyR1 in presence and absence of apoCaM (right panels). Although the density for apoCaM in the 2D class averages is subtle, the simulated projections show that this is expected.

Supplementary Tables

Supplementary Table 1. Cryo-EM map and model validation parameters

**Structure**	**Wt RyR1 EGTA (all classes, closed)**	**R615CRyR1 EGTA (all classes, open)**	**Wt RyR1 + CaM EGTA (class 1&2, closed)**	**Wt RyR1 + CaM EGTA (class 3, open)**	**R615CRyR1 + CaM EGTA (class 1, open)**	**R615CRyR1 + CaM EGTA (class 3, closed)**
**PDB ID**	6W1N	6X34	6X32	6X33	6X35	6X36
**EMDB ID**	EMD-21513	EMD-22017	EMD-22015	EMD-22016	EMD-22018	EMD-22019
**Data collection & processing**						
Micrographs	1,986	1,491	2,512		1949	
Magnification	59,000	59,000	75,000	75,000	75,000	75,000
Voltage (kV)	300	300	300	300	300	300
Electron exposure (e-/Å^2)	30	30	48	48	49	49
Defocus range (μm)	-1-3.5	-1.5-3.5	-1-2.5	-1-2.5	-1-2.5	-1-2.5
Pixel size (Å)	1.4	1.4	1.09	1.09	1.09	1.09
Symmetry imposed	C4	C4	C4	C4	C4	C4
Initial particle images (no.)	452,573	408,331	164,841	164,841	102,250	102,250
Final particle images (no.)	52,056	58,822	44,957	17,666	25,122	7,038
Map resolution (Å)[a] (FSC=0.143)	4.0	4.7	3.8	4.2	4.2	4.7
B-iso of Density Modified Map (Å^2)	32.9	36.1	32.7	34.9	34.2	50.8
**Refinement**						
Initial model used (PDB code)	5TB0	5TAW	6W1N	6W1N	6W1N	6W1N
Cross correlation (CC_{mask})	0.69	0.66	0.69	0.69	0.67	0.61
Model composition						
Non-hydrogen atoms	105312	97888	114312	112384	111876	100196
Protein residues	13996	13956	14752	14796	14736	13696
Ligands	Zn: 4	Zn: 4	Zn: 4	Zn: 4	Zn: 4	
B factor (Å^2)						
Protein	131.51	102.83	103.67	113.49	113.96	98.09
Ligand	220.91	174.53	150.48	186.07	188.81	
R.m.s. deviations						
Bond lengths (Å)	0.003	0.003	0.003	0.003	0.003	0.003
Bond angles (°)	0.876	0.896	0.847	0.865	0.836	0.841
Validation						
Molprobity score	1.25	1.16	1.39	1.11	1.13	1.34
Clashscore	4.78	3.70	3.27	3.20	3.37	3.28
CaBLAM (%)	1.13	2.78	1.05	1.70	2.44	2.32
Twisted Peptide	0	0	0	0	0	0
Poor rotomers (%)	0.32	0.61	0	0	0	0.05
Ramachandran plot						
Favored (%)	98.74	98.42	98.61	98.07	98.17	96.61
Allowed (%)	1.26	1.58	1.39	1.93	1.83	3.36
Disallowed (%)	0	0	0	0	0	0.03

[a] Resolution obtained from RELION 3.0-beta or CryoSPARC calculations prior to Phenix Resolve density modification, FSC threshold = 0.143.

Supplementary Table 2. Measured distances between wildtype (WtRyR1) and R615C (R615CRyR1) pRyR1 states with (+apoCaM) or without apo-calmodulin

	WtRyR1 Closed	R615CRyR1 Open	WtRyR1 +apoCaM Closed	WtRyR1 +apoCaM Open	R615CRyR1 +apoCaM Closed	R615CRyR1 +apoCaM Open
A-B Domain distance (Å) [a]	38	42	35	41	39	44
I4935 Cα distance from opposite subunits (Å)	10.9	17.4	10.7	17.0	11.5	16.3
Min. Pore diameter (Å)[b]	1.6	5.6	1.3	5.1	1.4	4.2
EF-EF (Å)[c]	120	122	120	135	127	131
NSol-BSol Angle (°) [d]	77.3	84.4	77.4	83.9	78.5	84.0

[a] Measured distances from the averaged A and B domain centers of neighbouring subunits

[b] Minimal diameters calculated from HOLE estimations

[c] Measured distances from the averaged EF-hand centers of opposite subunits

[d] Calculated angle from the Cα atoms of residues G409, I2168, and I2457 in one subunit.

SUPPLEMENTARY REFERENCE

1. Palovcak, E., Wang, F., Zheng, S.Q., Yu, Z., Li, S., Betegon, M., Bulkley, D., Agard, D.A. & Cheng, Y. A simple and robust procedure for preparing graphene-oxide cryo-EM grids. *J Struct Biol* **204**, 80-84 (2018).PMID: 30017701.
