# Supplementary material for: Pathological conformations of disease mutant Ryanodine Receptors revealed by cryo-EM
Source: Nat Commun. 2021 Feb 5;12:807. doi: 10.1038/s41467-021-21141-3 (PMC7864917; doi:10.1038/s41467-021-21141-3)
Supplement: Supplementary file 8 — Description of Additional Supplementary files [file 41467_2021_21141_MOESM8_ESM.docx]

Description of Additional Supplementary information

Title: Supplementary Movie S1

Description: Morph between WT and R615C pRyR1, both in the absence of CaM, showing the site of the mutation. The morph was generated by superposing the Nsol domains of both structures, highlighting the changes relative to this domain. The different domains are colored according to Figure 1a, with a neighboring subunit shown in gray. The mutation results in an increase of the angle between the Nsol and Bsol domains, which results in a disruption of interactions between NTD-B and NTD-A’ of neighboring subunits.

Title: Supplementary Movie S2

Description: Morph between WT and R615C pRyR1, both in the absence of CaM. The morph was generated by an overall superposition of the structures, highlighting the overall conformational changes in three different views. The different domains are colored according to Figure 1a.

Title: Supplementary Movie S3

Description: Structural changes in WT pRyR1 induced by binding of apoCaM. For the latter, the two different states (closed and open pRyR1+CaM) are shown. The different domains are colored according to Figure 1a. The animation shows the conformational changes in the N-lobe and C-lobe binding sites.

Title: Supplementary Movie S4

Description: Structural changes in R615C pRyR1 induced by binding of apoCaM. For the latter, the two different states (closed and open pRyR1) are shown. The different domains are colored according to Figure 1a. The animation shows the conformational changes of the Bsol region near the N-lobe, indicating that its conformation in the absence of CaM is different from either the open or closed R615C pRyR1 + apoCaM.

Title: Supplementary Movie S5

Description: Comparison of closed WT pRyR1+CaM and closed R615C pRyR1+CaM. The different domains are colored according to Figure 1a. Shown are changes near the mutation site, based on superposition of the Nsol domains. Next, changes are shown in three different views, based on overall superpositions.

Title: Supplementary Movie S6

Description: Comparison of open WT pRyR1+CaM and open R615C pRyR1+CaM. The different domains are colored according to Figure 1a. The animations are generated by overall superpositions, showing changes in three different views.
