# Supplementary material for: Pathological conformations of disease mutant Ryanodine Receptors revealed by cryo-EM
Source: Nat Commun. 2021 Feb 5;12:807. doi: 10.1038/s41467-021-21141-3 (PMC7864917; doi:10.1038/s41467-021-21141-3)
Supplement: Supplementary file 9 — Reporting Summary [file 41467_2021_21141_MOESM9_ESM.pdf]

nature research

Corresponding author(s): Filip Van Petegem

Last updated by author(s): Jan 4, 2021

Reporting Summary

Nature Research wishes to improve the reproducibility of the work that we publish. This form provides structure for consistency and transparency in reporting. For further information on Nature Research policies, see our Editorial Policies and the Editorial Policy Checklist.

Statistics

For all statistical analyses, confirm that the following items are present in the figure legend, table legend, main text, or Methods section.

n/a	Confirmed	
☐	☒	The exact sample size (*n*) for each experimental group/condition, given as a discrete number and unit of measurement
☐	☒	A statement on whether measurements were taken from distinct samples or whether the same sample was measured repeatedly
☐	☒	The statistical test(s) used AND whether they are one- or two-sided<br>*Only common tests should be described solely by name; describe more complex techniques in the Methods section.*
☒	☐	A description of all covariates tested
☐	☒	A description of any assumptions or corrections, such as tests of normality and adjustment for multiple comparisons
☐	☒	A full description of the statistical parameters including central tendency (e.g. means) or other basic estimates (e.g. regression coefficient) AND variation (e.g. standard deviation) or associated estimates of uncertainty (e.g. confidence intervals)
☒	☐	For null hypothesis testing, the test statistic (e.g. *F*, *t*, *r*) with confidence intervals, effect sizes, degrees of freedom and *P* value noted<br>*Give P values as exact values whenever suitable.*
☒	☐	For Bayesian analysis, information on the choice of priors and Markov chain Monte Carlo settings
☒	☐	For hierarchical and complex designs, identification of the appropriate level for tests and full reporting of outcomes
☒	☐	Estimates of effect sizes (e.g. Cohen's *d*, Pearson's *r*), indicating how they were calculated

Our web collection on statistics for biologists contains articles on many of the points above.

Software and code

Policy information about availability of computer code

Data collection	Thermo Fisher Scientific EPU software v2.6.0.59REL
Data analysis	GraphPad Prism v8.0; PyMOL v.2.3; PHENIX v.dev-3714 & v.1.18.2; Coot v0.9; UCSF Chimera v1.10; RELION v3.0; cryoSPARC v2.9, HOLE v9.0; UCSF pyem v0.5, Gctf v.1.05, Cryolo v1.3.6

For manuscripts utilizing custom algorithms or software that are central to the research but not yet described in published literature, software must be made available to editors and reviewers. We strongly encourage code deposition in a community repository (e.g. GitHub). See the Nature Research guidelines for submitting code & software for further information.

Data

Policy information about availability of data

All manuscripts must include a data availability statement. This statement should provide the following information, where applicable:

- Accession codes, unique identifiers, or web links for publicly available datasets
- A list of figures that have associated raw data
- A description of any restrictions on data availability

Raw cryo-EM and planar lipid bilayer data that was used in support of the findings are available from the corresponding author upon reasonable request. The authors declare that all other data supporting the findings of this study are available within the paper and its supplementary information files. The structures are available via the public repositories such as the protein data bank and the electron microscopy data bank via accession codes: 6W1N [https://doi.org/10.2210/pdb6W1N/pdb], 6X32 [https://doi.org/10.2210/pdb6X32/pdb], 6X33 [https://doi.org/10.2210/pdb6X33/pdb], 6X34 [https://doi.org/10.2210/pdb6X34/pdb], 6X35 [https://doi.org/10.2210/pdb6X35/pdb], and 6X36 [https://doi.org/10.2210/pdb6X36/pdb]. Source data for Fig 1b & 4a are available with this paper as an excel file.

Field-specific reporting

Please select the one below that is the best fit for your research. If you are not sure, read the appropriate sections before making your selection.

☒ Life sciences ☐ Behavioural & social sciences ☐ Ecological, evolutionary & environmental sciences

For a reference copy of the document with all sections, see nature.com/documents/nr-reporting-summary-flat.pdf

Life sciences study design

All studies must disclose on these points even when the disclosure is negative.

Sample size	No sample size calculation was performed. Sample size for each cryo-EM data set were determined on the basis for obtaining sufficient resolution maps for analysis. Sample size for functional experiments used was based on the level of variability within the replicates performed and previous experience with this method.
Data exclusions	No data was excluded in this study
Replication	All experiments are reproducible under the same condition. Small datasets were collected with ~500-1000 movies and pre processed to observe similar state for channel (open vs close) and to observe particle class distribution. Functional experiments were done with independent repeats using a new proteoliposome embedded into a fresh lipid bilayer for each independent recording.
Randomization	Protein conformations are effectively already randomized using single particle cryo-EM, as the method looks at snapshots of hundreds of thousands of randomly oriented particles embedded in vitreous ice. Thus further randomization of sample is not required as it is an intrinsic property of the method. No grouping was required for any of the experiments.
Blinding	No patients were involved in this study. Investigators were not blinded to group allocation, as no grouping was needed for this study.

Reporting for specific materials, systems and methods

We require information from authors about some types of materials, experimental systems and methods used in many studies. Here, indicate whether each material, system or method listed is relevant to your study. If you are not sure if a list item applies to your research, read the appropriate section before selecting a response.

Materials & experimental systems

n/a	Involved in the study	
☒	☐	Antibodies
☒	☐	Eukaryotic cell lines
☒	☐	Palaeontology and archaeology
☒	☐	Animals and other organisms
☒	☐	Human research participants
☒	☐	Clinical data
☒	☐	Dual use research of concern

Methods

n/a	Involved in the study	
☒	☐	ChIP-seq
☒	☐	Flow cytometry
☒	☐	MRI-based neuroimaging
